# Heterogeneous organophotocatalytic HBr oxidation coupled with oxygen reduction for boosting bromination of arenes

**DOI:** 10.1038/s41467-024-48349-3

**Published:** 2024-06-04

**Authors:** Jie Wang, Jiahao Liang, Hao Hou, Wei Liu, Hongru Wu, Hongli Sun, Wei Ou, Chenliang Su, Bin Liu

**Affiliations:** 1 International Collaborative Laboratory of 2D Materials for Optoelectronic Science & Technology, Engineering Technology Research Center for 2D Materials Information Functional Devices and Systems of Guangdong Province, Institute of Microscale Optoelectronics, Shenzhen University, Shenzhen, 518060 China; 2 State Key Laboratory of Radio Frequency Heterogeneous Integration, Shenzhen University, Shenzhen, 518060 China; 3 Department of Materials Science and Engineering, City University of Hong Kong, Tat Chee Avenue, Kowloon, Hong Kong, SAR 999007 China; 4 Department of Chemistry, Hong Kong Institute of Clean Energy (HKICE) & Center of Super-Diamond and Advanced Films (COSDAF), City University of Hong Kong, Tat Chee Avenue, Kowloon, Hong Kong, SAR 999007 China

**Keywords:** Photocatalysis, Synthetic chemistry methodology, Heterogeneous catalysis

## Abstract

Developing mild photocatalytic bromination strategies using sustainable bromo source has been attracting intense interests, but there is still much room for improvement. Full utilization of redox centers of photocatalysts for efficient generation of Br^+^ species is the key. Herein we report heterogenous organophotocatalytic HBr oxidation coupled with oxygen reduction to furnish Br_2_ and H_2_O_2_ for effective bromination of arenes over Al_2_O_3_ supported perylene-3,4,9,10-tetracarboxylic dianhydride (PTCDA). Mechanism studies suggest that O-vacancy in Al_2_O_3_ can provide Lewis-acid-type anchoring sites for O_2_, enabling unexpected dual-electron transfer from anchored photoexcited PTCDA to chemically bound O_2_ to produce H_2_O_2_. The in-situ generated H_2_O_2_ and Br_2_ over redox centers work together to generate HBrO for bromination of arenes. This work provides new insights that heterogenization of organophotocatalysts can not only help to improve their stability and recyclability, but also endow them with the ability to trigger unusual reaction mode via cooperative catalysis with supports.

## Abstract (web summary)

Developing mild photocatalytic bromination strategies using sustainable bromo source has been attracting intense interests, but there is still much room for improvement. Here, the authors report heterogenous organophotocatalytic HBr oxidation coupled with oxygen reduction to furnish Br_2_ and H_2_O_2_ for effective bromination of arenes.

## Introduction

Aryl bromides not only widely exist as pharmaceutical molecules, but also are essential building blocks for cross-coupling reactions in construction of valuable natural products and artificial molecules/materials^1–8^. Traditional strategies to make aryl bromides heavily relied on the use of Br_2_ and *N*-bromosuccinimide (NBS), which suffered from environmental-unfriendliness, poor functionalities-tolerance, over-bromination and low atom-efficiency^2,3,5,6^. Therefore, developing sustainable and effective bromination strategies is highly imperative. Recently, homogeneous organophotocatalytic bromination of (hetero)arenes using HBr or NaBr as the bromination reagent has been emerged as a promising strategy^9,10^. However, the low-reactivity, poor photostability and difficulty of reusing the organophotocatalysts significantly restrict their widespread applications. Therefore, on one hand, full utilization of redox centers for generation of Br^+^ species via Br^−^ oxidation by oxidative centers and reactive oxygen species (ROS) is crucial for improving reactivity^2^. On the other hand, heterogenization of advanced bromination organophotocatalysts not only helps to improve their photostability and recyclability, but also may introduce synergistic sites for cooperative catalysis.

Perylene-3,4,9,10-tetracarboxylic dianhydride (PTCDA) is a promising organic photoredox catalyst^11,12^ with proper oxidative potential (1.73 V vs. NHE, pH = 0) and reductive potential (−0.27 V vs. NHE, pH = 0) that may realize simultaneous oxidation of Br^−^ to Br^+^ and reduction of O_2_ to reactive oxygen species (ROS) for oxidative bromination^13^. Additionally, the conjugated perylene core and anhydride units of PTCDA shall enable its facile immobilization on support. As a result, in this work, PTCDA was chosen as a potential oxidative bromination organophotocatalyst. Unexpectedly, we discovered that PTCDA itself and physical mixture of PTCDA/metal oxide (i. e., Al_2_O_3_) showed little reactivity in organophotocatalytic oxidative bromination, even though superoxide radicals and Br_2_ were generated. However, when forming strong chemical interactions between PTCDA and Al_2_O_3_ (abbreviated as PTCDA/Al_2_O_3_) verified by in-situ diffuse reflectance infrared Fourier transform spectroscopy (DRIFTs), its reactivity for photocatalytic oxidative bromination was dramatically activated. Mechanistic studies disclosed that the anhydride units of PTCDA strongly interacted with the hydroxyl groups on the surface of Al_2_O_3_ upon high-temperature treatment. The O-vacancy in Al_2_O_3_ generated during high-temperature treatment provided Lewis-acid-type anchoring sites for O_2_. Upon light irradiation, the excited state catalyst was engaged in Br^−^ oxidation to Br_2_ and itself was converted to [cat]^• −^, which was subsequently quenched by donating an electron to O_2_ to furnish superoxide radical. Owing to the strong interaction between Al_2_O_3_ and O_2_, the formed superoxide radical could be fixed on the surface of Al_2_O_3_ that underwent another single electron reduction (SET) catalytic cycle to release H_2_O_2_. Finally, the in-situ generated Br_2_ and H_2_O_2_ could work together to produce HBrO for effective bromination of arenes.

## Results and discussion

### Heterogenization of PTCDA on Al_2_O_3_ support

**Fig. 1:**
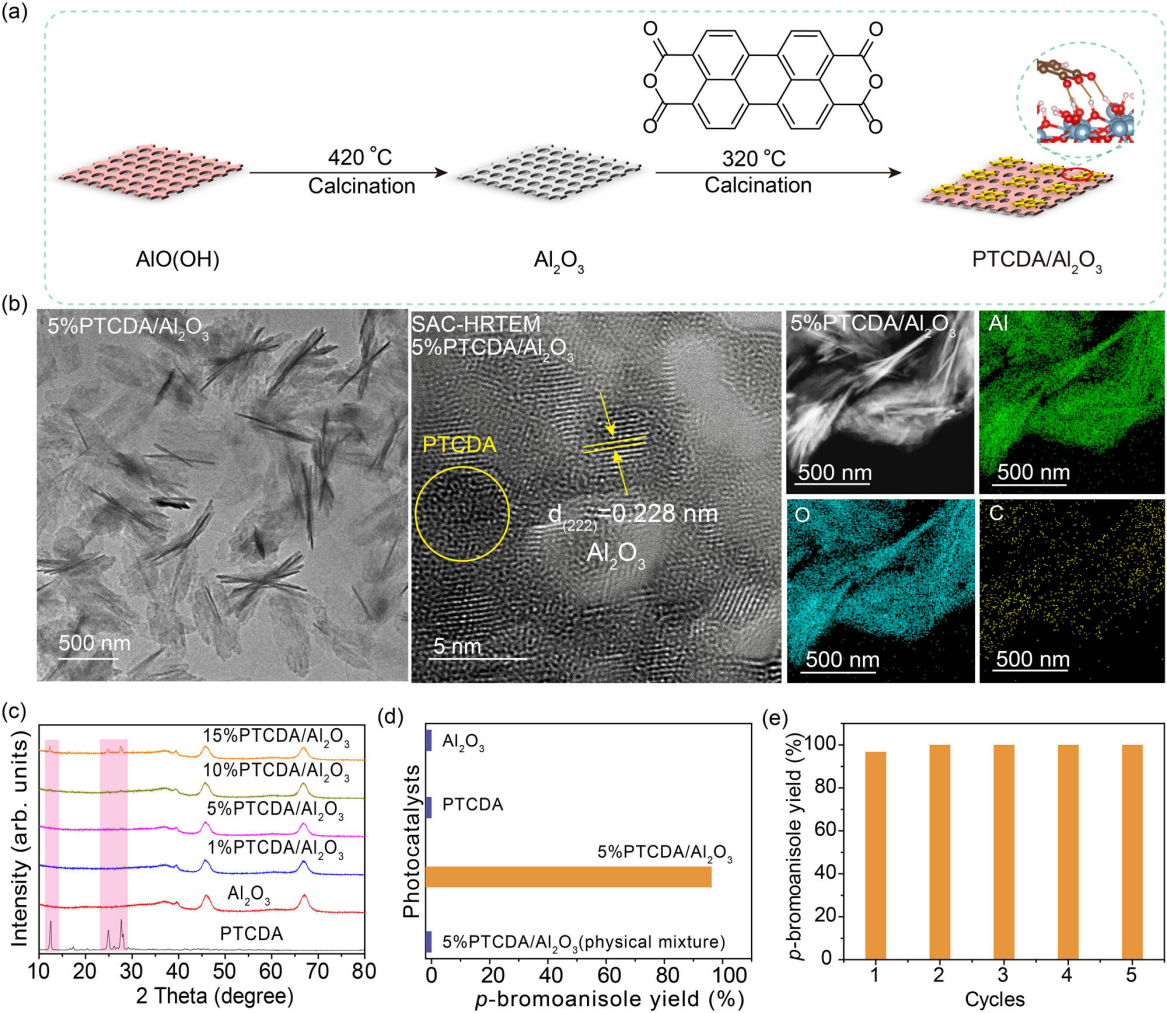
Synthesis, characterization and photocatalytic performance. **a** Schematic illustration showing the preparation procedure of PTCDA/Al_2_O_3_. **b** TEM and HRTEM images, scanning transmission electron microscope (STEM) image and the corresponding elemental mappings of 5%PTCDA/Al_2_O_3_. **c** XRD patterns of PTCDA/Al_2_O_3_ with different PTCDA contents. **d** Photocatalytic bromination reaction activities of Al_2_O_3_, PTCDA, 5%PTCDA/Al_2_O_3_(physical mixture), and 5%PTCDA/Al_2_O_3_. Reaction conditions: 10 mg of photocatalyst; temperature: 30 °C; reaction time: 5 h; acetonitrile: 1 mL; anisole: 0.2 mmol; HBr: 0.2 mL; O_2_: 1 atm; blue LED: 100 mW cm^−2^. **e** Evaluation of photocatalytic stability.

PTCDA has sufficient oxidative potential for Br^−^ oxidation (1.73 V vs. NHE, pH = 0) and reductive potential (−0.27 V vs. NHE, pH = 0) for O_2_ reduction^14–19^. To heterogenize PTCDA for providing Lewis-acid sites to adsorb organic molecules and molecular O_2_ to enhance catalytic activity and stability, Al_2_O_3_, an insulator, was selected as the potential cooperative support. PTCDA and two-dimensional (2D) Al_2_O_3_ nanosheets were evenly ground and calcined at 320 °C to establish strong interaction (Fig. 1a). Taking 5%PTCDA/Al_2_O_3_ (5 wt.% of PTCDA) as an example, transmission electron microscope (TEM) characterizations confirm the nanosheet morphology of Al_2_O_3_ and 5%PTCDA/Al_2_O_3_ (Fig. 1b and Supplementary Fig. 1a). Meanwhile, many channels are evenly observed in the HRTEM images of 5%PTCDA/Al_2_O_3_, similar to the structure of Al_2_O_3_ as shown in Supplementary Fig. 1b, c. Furthermore, through observation with high-resolution spherical aberration corrected TEM, PTCDA can be identified in 5%PTCDA/Al_2_O_3_, which is well-anchored on the surface of Al_2_O_3_. Besides, the 0.228 nm lattice spacings can be assigned to Al_2_O_3_ (222) plane, matching well with the X-ray diffraction (XRD) pattern (Fig. 1c)^20^. Energy dispersive X-ray spectroscopy (EDS) mappings show uniform distribution of the Al, C and O elements, demonstrating homogeneous attachment of PTCDA on Al_2_O_3_ surface. XRD patterns show gradual increase of PTCDA intensity with increasing PTCDA content in PTCDA/Al_2_O_3_ while the diffraction peaks of Al_2_O_3_ remain nearly constant.

To understand the support effect, Al_2_O_3_, PTCDA, their physical mixture, and PTCDA/Al_2_O_3_ were studied for bromination of arenes under visible light illumination and the results are shown in Supplementary Fig. 2 and Fig. 1d. PTCDA and PTCDA/Al_2_O_3_(physical mixture) show low reactivities in oxidative bromination reaction, while 5%PTCDA/Al_2_O_3_ displays excellent oxidative bromination performance with 95% yield (the actual mass percentage of PTCDA in 5%PTCDA/Al_2_O_3_ was determined to be around 5.0 wt.% by elemental analyzer as shown in Supplementary Table 1). More impressively, this organophotocatalyst shows excellent reusability and its reactivity can keep almost unchanged after multiple runs of bromination experiments (Fig. 1e and Supplementary Fig. 2).

### Interaction between PTCDA and Al_2_O_3_

**Fig. 2:**
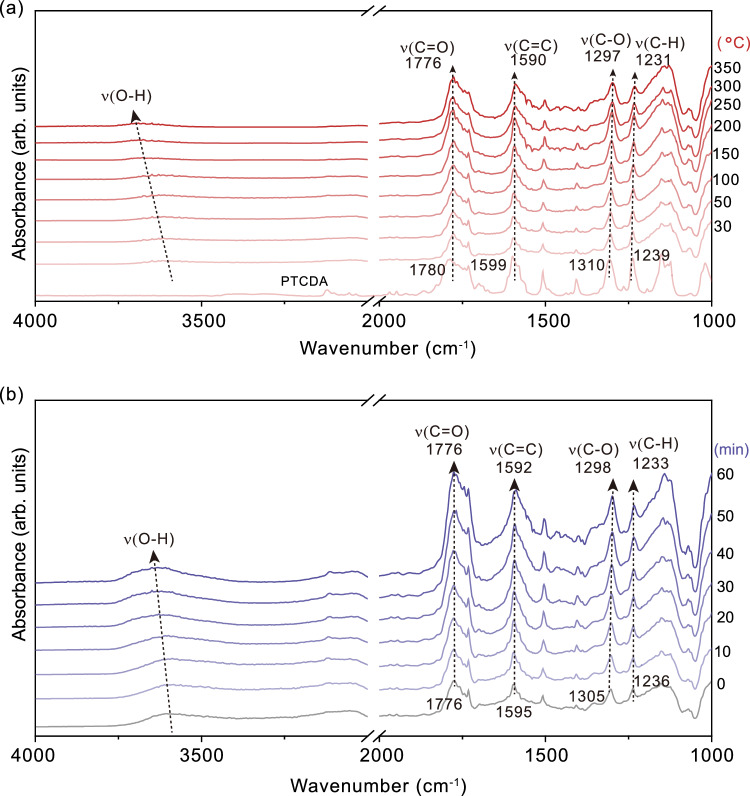
Interaction between PTCDA and Al_2_O_3_. **a** In-situ DRIFTs spectra of PTCDA and Al_2_O_3_ mixture calcined at different temperatures. **b** In-situ DRIFTs spectra of PTCDA and Al_2_O_3_ mixture calcined for different time durations.

To explore how simple heat treatment of PTCDA and Al_2_O_3_ mixture could dramatically improve photocatalytic bromination reaction performance, Fourier transform infrared (FTIR) spectroscopy and solid-state ^13^C NMR measurements were performed to investigate the structure change of PTCDA before and after calcination over Al_2_O_3_. As shown in Supplementary Fig. 3, the fingerprint peaks of PTCDA in both FTIR and CNMR spectra are well kept before and after thermal treatment but with slight shift, suggesting that PTCDA remained unchanged in structure after thermal treatment and formed strong interaction with Al_2_O_3_. Thermal gravimetry analysis (TGA) (Supplementary Fig. 4) confirms thermostability of PTCDA and PTCDA/Al_2_O_3_ below 400 °C. To gain more insights, in-situ diffuse reflectance infrared Fourier transform spectroscopy (DRIFTs) were conducted (Fig. 2). Fig. 2a shows that during calcination process, the vibration peaks of C=O, C=C, C‒O and C‒H of PTCDA apparently red shift, while the broad OH stretching vibration peak originated from Al_2_O_3_ significantly blue shifts; these vibration peaks are in great contrast to that of sole PTCDA (Supplementary Fig. 5a) or Al_2_O_3_ (Supplementary Fig. 5b), validating formation of strong interaction between PTCDA and surficial hydroxyl group on Al_2_O_3_^21^, which is further confirmed by heating PTCDA and Al_2_O_3_ mixture at 320 °C for different durations (Fig. 2b and Supplementary Fig. 5c).

**Fig. 3:**
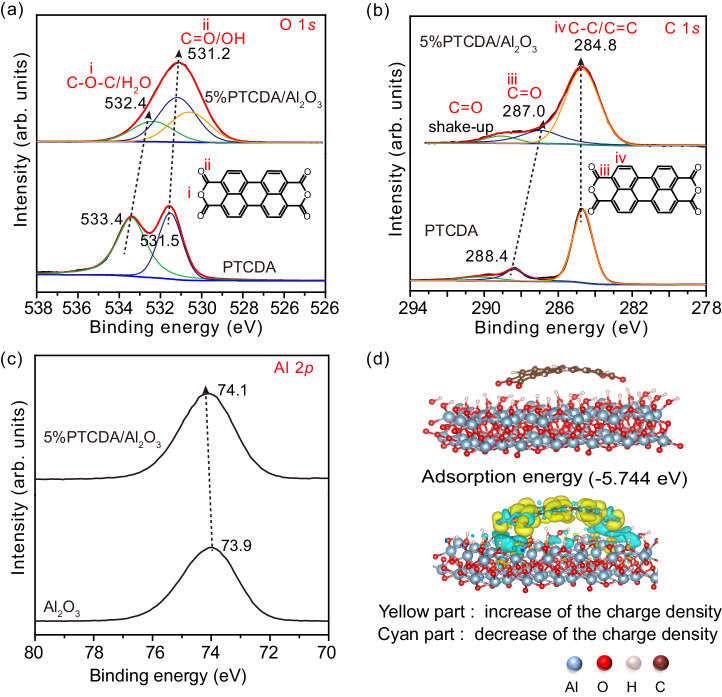
Anchoring mode between PTCDA on Al_2_O_3_ surface. High-resolution XPS spectra of (**a**) O 1*s* and (**b**) C 1 *s* for 5%PTCDA/Al_2_O_3_ and PTCDA. **c** High-resolution Al 2*p* XPS spectra for 5%PTCDA/Al_2_O_3_ and Al_2_O_3_. **d** The adsorption mode of PTCDA on Al_2_O_3_ surface and the differential charge of PTCDA on Al_2_O_3_ surface. The isovalue is 1.25 × 10^−3^ eÅ^−3^. The yellow/cyan region represents increase/decrease in charge density.

The interaction mode of PTCDA and Al_2_O_3_ was further characterized by X-ray photoelectron spectroscopy (XPS) (Fig. 3a, b). After calcining PTCDA with Al_2_O_3_, the O 1*s* binding energy of C=O and C‒O in PTCDA slightly shifted from 531.5 to 531.2 eV and from 533.4 to 532.4 eV, respectively^22^. The C 1*s* binding energy of C=O in PTCDA accordingly shifted from 288.4 to 287.0 eV^16^, but one of the C 1 *s* for C‒C/C=C bond had little change. Moreover, the binding energy of Al 2*p* shifted from 73.9 to 74.1 eV (Fig. 3c)^20^. These results indicate that the O or C charge density of C‒O and C=O increases while the Al charge density decreases after forming a strong interaction between PTCDA and Al_2_O_3_ via thermal treatment, which can be attributed to the electron withdrawing effect of anhydride units in PTCDA. It also suggests that the interaction between PTCDA and Al_2_O_3_ is mainly formed via O of C‒O and C=O in PTCDA with surface OH on Al_2_O_3_. To further probe the electron state, the PTCDA adsorption configuration on Al_2_O_3_ was calculated and differential charge analysis is performed (Fig. 3d). After attached on Al_2_O_3_ surface, PTCDA molecule exhibited an increase in charge density, which is consistent with the results obtained by XPS. Meanwhile, other adsorption modes, differential charge and Bader charge as well as negative-projected crystal orbital Hamilton population (−pCOHP) for the O and H atoms with the shortest bond length were also investigated (Supplementary Fig. 6). The calculated adsorption energy is −4.808 eV for model 2 and −3.356 eV for model 3, more positive than that for model 1 (−5.744 eV), and the integral COHP values are −2.13324 for model 1, −1.85124 for model 2, and −1.44866 for model 3. These results indicate the strongest interaction between PTCDA and Al_2_O_3_ in model 1.

Supplementary Fig. 7a compares the UV-vis absorption spectra for PTCDA, Al_2_O_3_, 5%PTCDA/Al_2_O_3_(physical mixture), and 5%PTCDA/Al_2_O_3_. It can be found that the absorption spectrum of 5%PTCDA/Al_2_O_3_ blue shifted in the visible range, while there was almost no change for the absorption spectrum of 5%PTCDA/Al_2_O_3_(physical mixture) as compared to that of PTCDA. Since Al_2_O_3_ only absorbs UV light (<400 nm)^23^, the blueshift of light absorption for 5%PTCDA/Al_2_O_3_ might result from hybridization of electronic orbitals formed between PTCDA and Al_2_O_3_. The absorption edge of PTCDA and 5%PTCDA/Al_2_O_3_(physical mixture) are at around 639 nm (bandgap: 1.94 eV) and 631 nm (bandgap: 1.96 eV), respectively^17^, while the absorption edge of 5%PTCDA/Al_2_O_3_ is at about 681 nm (bandgap: 1.82 eV). According to the published data^17,24,25^, the reduction potential of PTCDA is at −0.27 V vs. NHE (pH = 0) and the conduction band edge of Al_2_O_3_ is at −0.30 V vs. NHE (pH = 0), which are more negative than the potential of O_2_/O_2_^•−^ [−0.13 V vs. NHE (pH = 0)] and O_2_/O_2_^2−^ [0.68 V vs. NHE (pH = 0)], implying that 5%PTCDA/Al_2_O_3_(physical mixture) and 5%PTCDA/Al_2_O_3_ are able to realize O_2_ activation and thus produce ROS under light illumination (Supplementary Fig. 7b). To further understand the O_2_ activation process, femtosecond transient absorption spectroscopy spectra were recorded under the condition of air saturation (Supplementary Fig. 7c, d)^26^. There shows an obvious broad positive signal in the wavelength range from 550 to 750 nm over 5%PTCDA/Al_2_O_3_ (Supplementary Fig. 7c). As time went from 0 to 1 ps, the intensity of the positive signal gradually increased to a maximum. Then, the signal intensity slightly decreased at t = 10 ps and maintained stable during 10–30 ps. Afterwards, the signal gradually reduced to 0 in the time range from 30 to 1000 ps. The increase of the positive signal results from accumulation of photogenerated electrons in the excited states. After reaching maximum, the slight signal intensity decrease (1–10 ps) and short maintenance of balanced states (10–30 ps) can be attributed to O_2_ activation by the excited electrons. The signal in the wavelength range from 550 to 750 nm was much weaker over 5%PTCDA/Al_2_O_3_(physical mixture) (Supplementary Fig. 7d).

### Photocatalytic mechanism

Control experiments under different reaction atmospheres pinpoints the important role of O_2_ for photocatalytic bromination of arenes over PTCDA/Al_2_O_3_ (Supplementary Table 2, entry 1-2). To identify the key reactive species, scavenger studies were carefully designed and carried out, butylated hydroxytoluene (BHT), nitrotetrazolium blue chloride (NBT), *t*-butyl alcohol (*t*-BuOH) and 9,10-diphenylanthrene (DPA) are used as the sacrificial agents to probe the roles of carbon radical (R•), superoxide radical (•O_2_^−^), hydroxyl radical (•OH) and singlet oxygen (^1^O_2_), respectively^27^, and the results are shown in Supplementary Fig. 8a. When NBT or *t*-BuOH was added into the reaction system, the yield of *p*-bromoanisole decreased from 95% to 28% or 43%, indicating that •O_2_^−^ and •OH played critical roles in the photocatalytic bromination reaction. On the other hand, addition of BHT or DPA had little influence on the photocatalytic performance. To directly probe the ROS, electron paramagnetic resonance (EPR) experiments were conducted. Under light irradiation with continuous bubbling of O_2_ (Supplementary Fig. 8b), intense EPR signals of •O_2_^−^ were observed over PTCDA, 5%PTCDA/Al_2_O_3_ and 5%PTCDA/Al_2_O_3_(physical mixture), but not over Al_2_O_3_^28^. In consideration of the low reactivities of PTCDA and PTCDA/Al_2_O_3_(physical mixture) as shown in Fig. 1d, based on the results of scavenger studies, it is concluded that •O_2_^−^ species is essential but not the only prerequisite for the photocatalytic bromination reaction. •O_2_^−^ might work as an intermediate to generate other ROS, for example H_2_O_2_ can be generated via SET reduction of •O_2_^−^. 0.009 mmol of H_2_O_2_ (quantified by iodometry method) was observed without adding HBr as the bromine source in the reaction system of 5%PTCDA/Al_2_O_3_ (Supplementary Table 3, entry 1), while no H_2_O_2_ could be detected in the reaction system of PTCDA and 5%PTCDA/Al_2_O_3_(physical mixture) (Supplementary Table 3, entry 2-3). To investigate the role of H_2_O_2_ in oxidative bromination reaction with HBr, 0.25 mmol of H_2_O_2_ was purposely added into the reaction system in the absence of photocatalyst and light, 96% yield of *p*-bromoanisole was obtained (Supplementary Table 2, entry 5), suggesting that H_2_O_2_ worked together with HBr in the oxidative bromination of arenes. H_2_O_2_ can be generated via 2e^−^ photocatalytic oxygen reduction reaction or 2e^−^ photocatalytic water oxidation reaction^29–35^. To verify the reaction pathway of H_2_O_2_, electron scavenger (H_2_PtCl_6_) was added into the reaction system under Ar atmosphere, and no H_2_O_2_ could be detected, which excludes the 2e^−^ photocatalytic water oxidation pathway to generate H_2_O_2_. Control experiments under O_2_ and Ar atmosphere as shown in Supplementary Table 2 (entries 1-2) and EPR studies as shown in Supplementary Fig. 9 verify that the H_2_O_2_ was produced via oxygen reduction reaction during the photocatalytic bromination reaction.

**Fig. 4:**
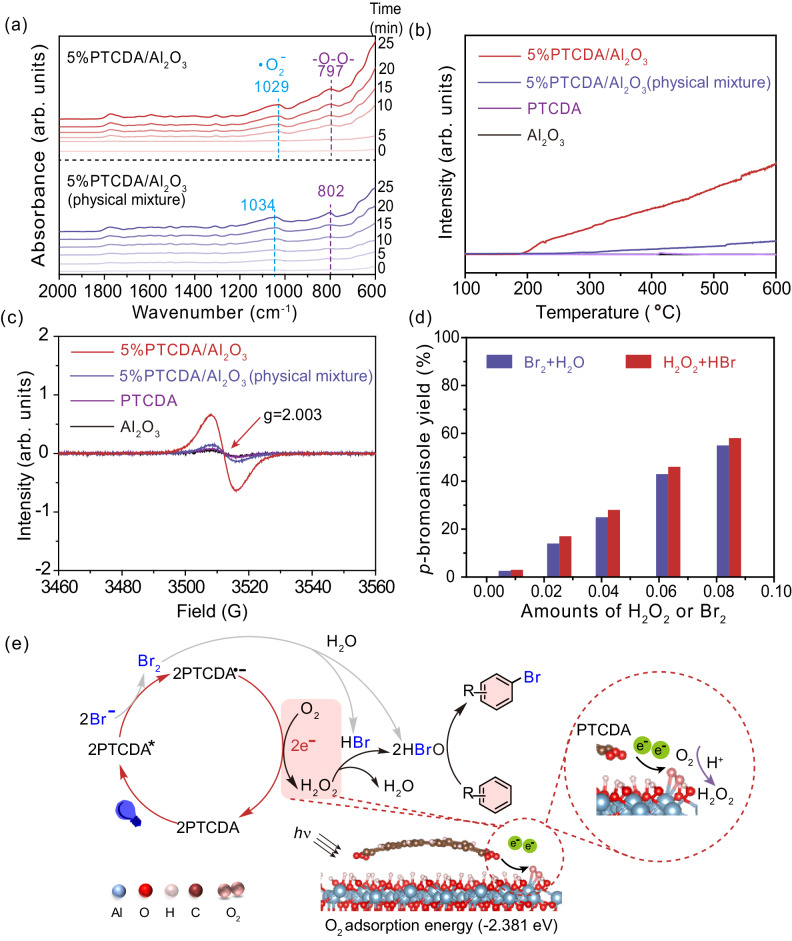
Photocatalytic mechanism. **a** In-situ DRIFTs spectra recorded over 5%PTCDA/Al_2_O_3_ and 5%PTCDA/Al_2_O_3_(physical mixture) under visible light irradiation for different time durations. **b** O_2_-TPD and **c** EPR spectra of Al_2_O_3_, PTCDA, 5%PTCDA/Al_2_O_3_(physical mixture), and 5%PTCDA/Al_2_O_3_. **d** The influence of H_2_O_2_ or Br_2_ on photocatalytic bromination reaction. temperature: 30 °C; reaction time: 5 h; acetonitrile: 1 mL; anisole: 0.2 mmol; Air: 1 atm; blue LED: 100 mW cm^−2^. In the system of Br_2_ + H_2_O, HBr was not added, but in H_2_O_2_ system, HBr was added. The amounts of H_2_O_2_ or Br_2_ added are 0.0042, 0.0084, 0.0253, 0.0422, 0.0633, and 0.0843 mmol, respectively. **e** The possible photocatalytic bromination reaction mechanism.

Most organophotocatalytic reactions undergo oxidative and reductive quenching cycles via single electron transfer (SET) processes. Herein we discover that the heterogeneous PTCDA/Al_2_O_3_ can enable a different dual-electron transfer process to generate H_2_O_2_ instead of the traditional SET. To shed light on this unusual phenomenon, in-situ DRIFTs was performed to probe the oxygen reduction reaction under visible light irradiation^36,37^. As shown in Fig. 4a, adsorption of •O_2_^−^ at 1029 cm^−1^ can be clearly observed over 5%PTCDA/Al_2_O_3_(physical mixture). Interestingly, the -O-O- peak at 797 cm^−1^ is more intensive than the •O_2_^−^ peak over 5%PTCDA/Al_2_O_3_. These results suggest that the O_2_ reduction behavior over 5%PTCDA/Al_2_O_3_ is different from that over PTCDA and 5%PTCDA/Al_2_O_3_(physical mixture). To experimentally probe O_2_ adsorption, O_2_-TPD (temperature programmed desorption) experiments were conducted. As shown in Fig. 4b, the O_2_ adsorption capacity (chemical adsorption) over 5%PTCDA/Al_2_O_3_ is much higher than that over PTCDA and 5%PTCDA/Al_2_O_3_(physical mixture). EPR measurements suggest a much higher concentration of oxygen vacancies in 5%PTCDA/Al_2_O_3_ (Fig. 4c), which were possibly generated during the heat-treatment of Al_2_O_3_ and PTCDA (demonstrated in Supplementary Fig. 10a, b). The rich surface oxygen vacancies on 5%PTCDA/Al_2_O_3_ shall provide abundant adsorption sites to chemically activate O_2_ molecules, thus promoting two-electron transfer from the firmly anchored photoexcited PTCDA to chemically bound O_2_ under light irradiation to produce H_2_O_2_. Theoretical calculations further support enhanced O_2_ adsorption on PTCDA/Al_2_O_3_(Model 1) (ΔG_*O2_ = −2.381 eV, Fig. 4e) as compared to that on PTCDA/Al_2_O_3_(physical mixture, Model 3) (ΔG_*O2_ = −1.735 eV, Supplementary Fig. 10c).

Next the contributions of H_2_O_2_ and Br_2_ in oxidative bromination reaction were studied. According to literature^2,5,38^, H_2_O_2_ is able to oxide HBr to generate active electrophilic HBrO. Br_2_ can react with water to form HBrO as well. The HBrO can subsequently react with the substrate in an electrophilic substitution reaction. First, the generation of Br_2_ was well demonstrated by conducting the photocatalytic reaction in the presence of electron scavenger (H_2_PtCl_6_) under Ar atmosphere, during which HBr oxidation and generation of Br_2_ (at 380 nm) could be clearly observed in the UV-vis absorption spectra (Supplementary Fig. 11a, b). Then, H_2_O_2_ or Br_2_ was directly added into the reaction system in absence of light and photocatalyst to investigate their roles in oxidative bromination. As shown in Fig. 4d, with increasing amount of added H_2_O_2_ or Br_2_, the yield of *p*-bromoanisole increases. Meanwhile, to verify the role of HBrO, HBrO was purposely added into the reaction system without photocatalyst in dark. Addition of 0.25 mmol HBrO resulted in a *p*-bromoanisole yield of 95%, confirming the contribution of HBrO in the bromination reaction (Supplementary Table 2, entry 6). With these results, it is concluded that the major roles of the photoredox centers in PTCDA/Al_2_O_3_ are to furnish Br_2_ and H_2_O_2_, which work synergistically to generate active electrophilic HBrO for sequential bromination.

Figure 4e proposes the possible photocatalytic bromination reaction mechanism over PTCDA/Al_2_O_3_ photocatalyst. Under light irradiation, the excited state PTCDA* oxidizes Br^−^ to produce Br_2_ and PTCDA^•−^ via a SET process^18^. The resultant PTCDA^•−^ undergoes a reductive quenching cycle with O_2_ to regenerate PTCDA, meanwhile O_2_ obtains one electron and one proton to form •OOH, which is firmly fixed on the surface of photocatalyst. Next, the •OOH takes one more electron and one more proton to make H_2_O_2_. According to the reported literatures and our experimental data^2,5^, the reaction between Br_2_ and water or H_2_O_2_ and Br^−^ can produce active electrophilic HBrO species, which will spontaneously react with arenes to produce brominated products.

### Substrate scopes

**Fig. 5:**
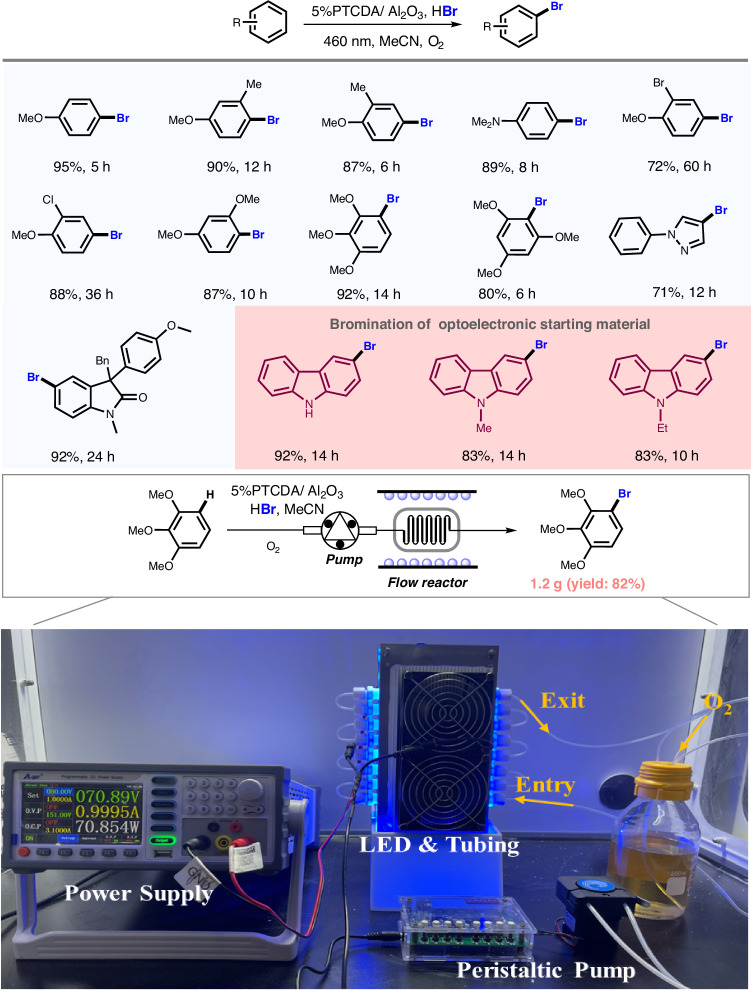
The expanding of substrate scope for photocatalytic bromination and the implementation of fluid-phase reaction using 5%PTCDA/Al_2_O_3_ as a photocatalyst. The yields of brominated products with anisole, *o*-methylanisole and *m*-methylanisole as substrates are quantified by gas chromatography-mass spectrometer (GC-MS) with *p*-fluoronitrobenzene as the internal standard. The yields of other brominated products are quantified by isolated yield. Fluid-phase reaction conditions: room temperature; time: 4 h; acetonitrile: 90 mL; substrate: 1 g; HBr: 10 mL; O_2_: 1 atm; blue LED:50 mW cm^−2^; and flowrate: 10 mL min^−1^.

The photocatalytic oxidative bromination reaction developed here shows good extensibility to a large family of arenes (Fig. 5)^39–46^. Electron-rich arenes bearing one, two and three substituents are found to be well tolerated with corresponding products in good to excellent yields. Bromination of heteroarenes has also been demonstrated by using 1-phenylpyrazole as the model substrate, giving the desired product in 71% yield. Arenes with sensitive functional groups including secondary amine, tertiary amine, halides and heterocycles are compatible with the photocatalytic oxidative bromination reaction system. Taking carbazoles as substrates, their brominated products, which can be used as building blocks for making organic photoelectronic materials, could be obtained in 80–92% yields. For the purpose of practical application, a gram-scale flow system^4,47^ using 1,2,3-trimethoxybenzene as the model substrate was designed as shown in Fig. 5, which could produce brominated product in 82% yield.

In summary, we have demonstrated that heterogenization of organophotocatalyst such as PTCDA can dramatically switch on the reactivity in photocatalytic oxidative bromination reaction using HBr as the atom-economic bromination reagent. This mild photocatalytic bromination strategy shows good extensibility, recyclability and scalability. Mechanism studies discover that heat treatment of PTCDA and Al_2_O_3_ can not only establish strong chemical interactions between them to facilitate electron transfer, but also introduce O vacancies on Al_2_O_3_ to adsorb molecular O_2_. Benefiting from these synergistic effects, an unusual dual-electron transfer reaction mode is newly presented, where O_2_ is reduced to H_2_O_2_ on the photoreductive centers. Meanwhile, HBr oxidation occurs on photooxidative centers to furnish Br_2_. Both Br_2_ and H_2_O_2_ contribute for the effective bromination of arenes. This work highlights that heterogenization of organophotocatalysts exhibits great potential for improving their stability and recyclability, and more importantly, it is able to trigger different reaction mode via synergistic catalysis with the well-designed supports.

## Methods

### Chemicals

Aluminum nitrate nonahydrate (Al(NO_3_)_3_•9H_2_O), urea, 3,4,9,10-perylenetetracarboxylic dianhydride (PTCDA, 98%), triethylamine hydrochloride, anisole, acetonitrile, and hydrogen bromide (HBr, 48% in H_2_O) were purchased from Energy Chemical and used without further purification.

### Preparation of 2D Al_2_O_3_ nanosheets

2D Al_2_O_3_ nanosheets were prepared through a hydrothermal method^21,48,49^. In a typical synthesis, 4.5 g of Al(NO_3_)_3_•9H_2_O and 5.1 g of urea were added to a 250 mL round bottom flask, followed by adding 200 ml of ultrapure water. Then the obtained mixture was maintained at 150 °C for 24 h. After cooling, the white product was washed to neutral using ultrapure water, and then dried in an oven at 75 °C for 24 h to obtain AlO(OH). AlO(OH) was calcined under air atmosphere for 8 h at 420 °C to make Al_2_O_3_ nanosheets.

### Preparation of PTCDA/Al_2_O_3_

For a typical synthesis of 5%PTCDA/Al_2_O_3_, 0.3 g of Al_2_O_3_ and 0.0158 g of PTCDA were ground evenly and then added into a quartz boat, which was calcined at 320 °C for 1 h inside a tube furnace under air atmosphere at a heating rate of 5 °C/min. After cooling down to room temperature, the obtained sample was washed twice with 0.1 M triethylamine hydrochloride solution, followed by three times with ultrapure water and once with ethanol, and then dried at 60 °C in a vacuum oven for 12 h to obtain a composite of PTCDA and Al_2_O_3_, labeled as 5%PTCDA/Al_2_O_3_. PTCDA/Al_2_O_3_ with different PTCDA mass percentages were obtained using the same method by only changing the mass of PTCDA.

### Characterizations

Transmission electron microscopy (TEM) measurements were carried out on a JEOL F200 microscope with accelerating voltage of 200 kV. The high-resolution transmission electron microscope images were obtained on a JEOL JEM-ARF200F TEM/STEM equipped with a spherical aberration corrector. The XRD patterns were recorded on a Rigaku Ultima IV X-RAY diffractometer with Cu Kα radiation (λ = 1.5418 Å) in the 2*θ* range from 5 to 80° at a scanning rate of 8° min^−1^. X-ray photoelectron spectroscopy (XPS) and in-situ XPS under light irradiation were conducted on a Escalab 250Xi spectrometer at room temperature using an Al Kα X-ray source (*hν* = 1486.6 eV). The C 1*s* peak at 284.8 eV was used as the reference for the calibration of the binding energy. UV-vis diffuse reflectance spectra were measured on an Agilent Technologies Cary Series UV-vis-NIR spectrometer. In-situ diffuse reflectance infrared Fourier transform spectroscopy (DRIFTs) measurements were conducted on a Bruker Tensor II spectrometers. The percentage of PTCDA was determined by an organic element analyzer on elementar EL III. Thermogravimetric/differential thermal (TG/DTA) analysis was carried out on a Thermo plus EV2 in air with a heating rate of 10 °C min^−1^. ^1^H and ^13^C NMR spectra were recorded by a Bruker AVANCE III spectrometer (frequencies of 600 and 100 MHz) and the chemical shift was referenced to TMS (tetramethylsilane).

### Photocatalytic bromination reaction

Typically, 10 mg of photocatalyst, 1 mL of anisole in acetonitrile solution (0.2 M) and 0.2 mL of HBr solution were added into a 10 mL vial. The vial was then purged with O_2_ for 1 min to maintain the O_2_ pressure at 1 atm and kept at 30 °C in an oil bath for 5 h under visible light irradiation [Blue LED (center wavelength, 460 nm), 100 mW cm^−2^]. Afterwards, 0.01 mmol of *p*-fluoronitrobenzene as the internal standard and 3 mL of ultrapure water were added to the vial. The products were extracted using 1 mL of ethyl acetate. The supernatant was analyzed using Agilent Technologies 7820 gas chromatography equipped with a WondaCap 5 column. The amounts of products and reactants were calculated using an internal standard method.

### H_2_O_2_ production and detection

The H_2_O_2_ concentration in solution was determined by an iodometry method as reported^50^. After reaction, 0.1 mL of reaction solution was centrifuged, followed by adding 1 ml of potassium titanium oxalate solution (0.1 mol L^−1^), 1 mL of KI solution (0.4 M) and 4 ml of deionized water. The concentration of H_2_O_2_ was determined based on the absorbance at 350 nm in the UV-vis spectra.

### EPR experiments

To capture •O_2_^−^, 10 mg of photocatalyst, 1 mL of methanol, and 50 μL of 5,5-dimethyl-1-pyrroline N-oxide (DMPO) solution (methanol: DMPO = 10: 1) were added into a photocatalytic reactor and then irradiated by visible light under O_2_ atmosphere. 20 μL of solution was taken out for EPR measurement after different durations of photocatalytic reaction. To capture •OH, 10 mg of photocatalyst, 1 mL of H_2_O, 0.2 mmol of H_2_PtCl_6_ and 50 μL of 5,5-dimethyl-1-pyrroline N-oxide (DMPO) solution (acetonitrile: DMPO = 10: 1) were added into a photocatalytic reactor and then irradiated by visible light under Ar atmosphere. 20 μL of solution was taken out for EPR measurement after different durations of photocatalytic reaction. Room-temperature EPR spectra were recorded on Bruker EMXPLUS10/12 EPR electron paramagnetic resonance spectrometer.

### TPD experiments

Oxygen desorption was measured by temperature programmed desorption (TPD) of O_2_ in a micro-reactor. Typically, 100 mg of catalyst was added to a micro-reactor, pre-treated in Ar at 200 °C for 1 h and then cooled to 30 °C. The adsorption of O_2_ (30 mL min^−1^) was implemented for 10 min at 30 °C and then the catalyst was flushed with Ar (30 mL min^−1^) for 30 min at 30 °C to remove the physically adsorbed gas on the surface of the catalyst. Programmed desorption was performed at a heating rate of 10 °C min^−1^ from 50 to 800 °C probed by a TCD detector.

### Computational details

The spin-polarized density functional theories (DFT) were carried out by using the Vienna Ab initio Simulation Package (VASP)^51^. (110) surface was chosen as the active surface to represent the as-prepared γ-Al_2_O_3_ in our calculation model because that it is estimated that (110) surface is the predominant exposed-surface according to the Gibbs–Curie–Wulff law^52^, which occupies 74% of the total surface area, followed by (100) surface (16%) and (111) surface (10%). The Perdew-Burke-Ernzerhof generalized-gradient approximation functional was used to describe the interaction between electrons^53^. The DFT-D2 method was adopted to evaluate the van der Waals (vdW) interaction^54^. The energy cutoff was set to 400 eV. The energy criterion was set to 10^−5^ eV in the iterative solution of the Kohn-Sham equation. The Brillouin zone integration was performed using a 2 × 2 × 1 k-mesh. All the structures are relaxed until the residual forces on the atoms have declined to less than 0.02 eV/Å.

### Supplementary information

Supplementary Information

Peer Review File

## Data Availability

The data generated in this study are provided in the article and the Supplementary Information file and are available from the corresponding author upon request. Source data are kept in the repositories 10.6084/m9.figshare.25669848.
