# Supplementary material for: Heterogeneous organophotocatalytic HBr oxidation coupled with oxygen reduction for boosting bromination of arenes
Source: Nat Commun. 2024 Jun 4;15:4744. doi: 10.1038/s41467-024-48349-3 (PMC11150450; doi:10.1038/s41467-024-48349-3)
Supplement: Supplementary file 1 — Supplementary Information [file 41467_2024_48349_MOESM1_ESM.pdf]

Supplementary Information

Heterogeneous Organophotocatalytic HBr Oxidation Coupled with Oxygen Reduction for Boosting Bromination of Arenes

Jie Wang[1,2], Jiahao Liang[1,2], Hao Hou[1,2], Wei Liu[1,2], Hongru Wu[1,2], Hongli Sun[1,2], Wei Ou[1,2], Chenliang Su[1,2*] and Bin Liu[3,4*]

[1]International Collaborative Laboratory of 2D Materials for Optoelectronic Science & Technology, Engineering Technology Research Center for 2D Materials Information Functional Devices and Systems of Guangdong Province, Institute of Microscale Optoelectronics, Shenzhen University, Shenzhen 518060, China.

[2]State Key Laboratory of Radio Frequency Heterogeneous Integration, Shenzhen University, Shenzhen 518060, China

[3]Department of Materials Science and Engineering, City University of Hong Kong, Tat Chee Avenue, Kowloon, Hong Kong SAR 999007, China.

[4]Department of Chemistry, Hong Kong Institute of Clean Energy (HKICE) & Center of Super-Diamond and Advanced Films (COSDAF), City University of Hong Kong, Tat Chee Avenue, Kowloon, Hong Kong SAR 999007, China.

[*]Email: chmsuc@szu.edu.cn; bliu48@cityu.edu.hk

1. Figures and Tables

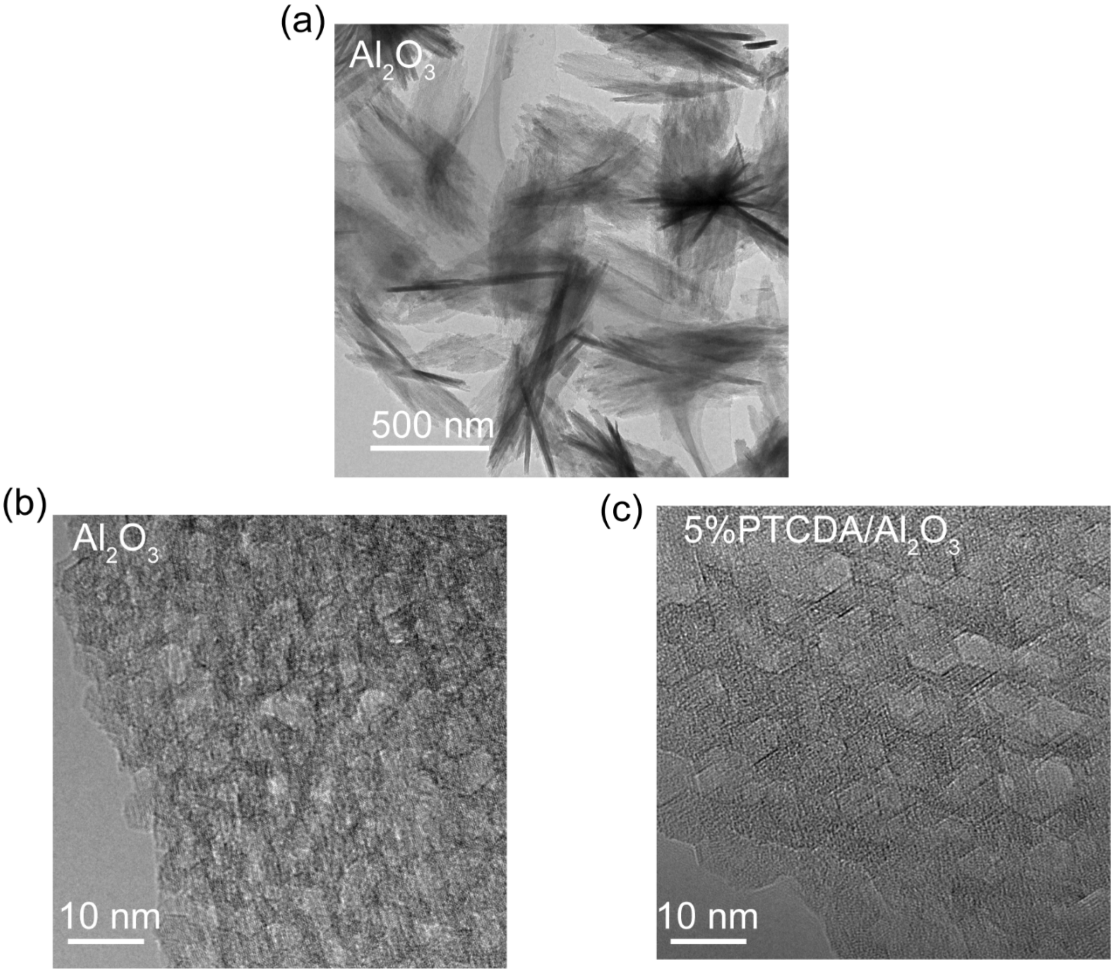

Supplementary Figure 1. Morphology characterizations. (a) TEM image of Al_2O_3 and (b-c) HRTEM images of Al_2O_3 and 5%PTCDA/Al_2O_3.

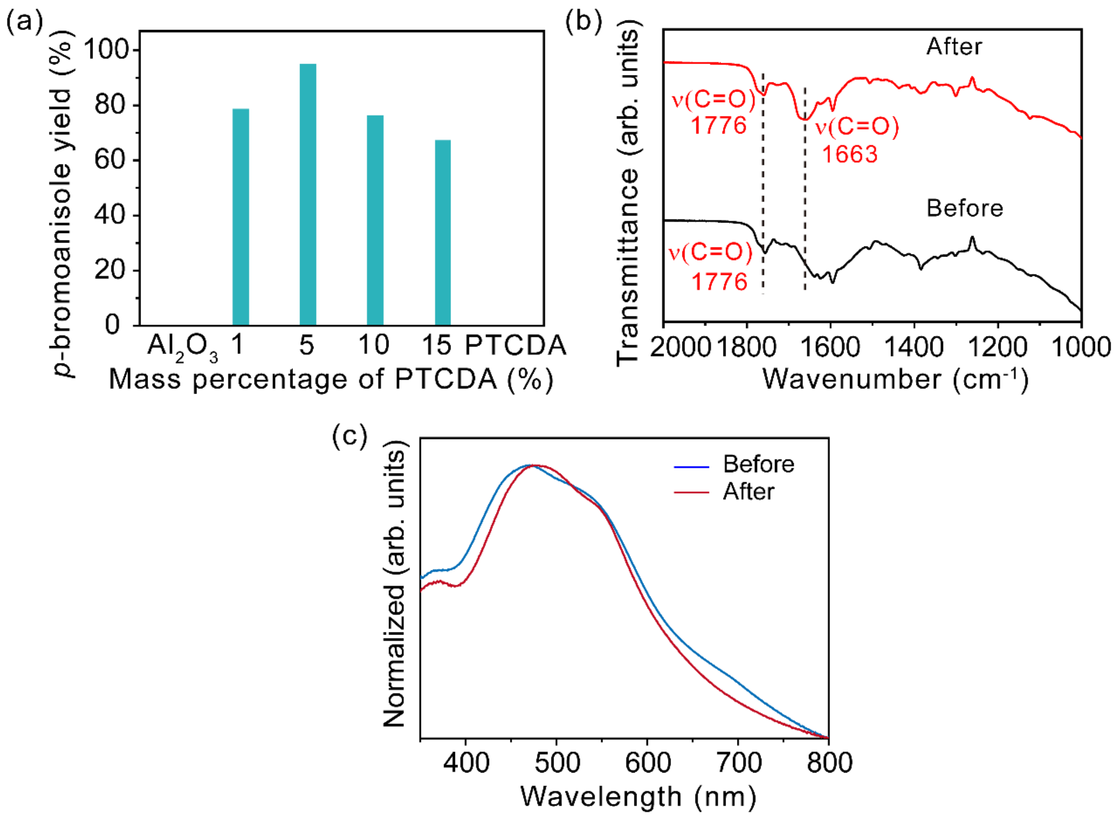

Supplementary Figure 2. Photocatalytic performance. (a) Photocatalytic performance of PTCDA/Al_2O_3 with different PTCDA mass percentages. Reaction conditions: photocatalyst: 10 mg; temperature: 30 °C; time: 5 h; acetonitrile: 1 mL; anisole: 0.2 mmol; HBr: 0.2 mL; O_2: 1 atm; blue LED: 100 mW cm^{-2}. (b) FTIR and (c) UV-vis absorption spectra of 5%PTCDA/Al_2O_3 before and after 5 cycles of photoreaction.

After five photocatalytic cycles, it was found that the yield of *p*-bromoanisole still exceeded 90%. There showed a new obvious infrared absorption peak at 1663 cm^{-1}, which could be attributed to the stretching vibration of C=O bond, due to partial hydrolysis of anhydride in the presence of HBr.[1] This did not change the light absorption property of 5%PTCDA/Al_2O_3 (Supplementary Figure 2c) as light absorption of PTCDA is mainly resulted from perylene rings. Hence, 5%PTCDA/Al_2O_3 exhibited stable photocatalytic activity.

Supplementary Table 1. Actual mass percentage of PTCDA in PTCDA/Al_2O_3 obtained from elemental analyzer.

Entry	Catalysts	Measured mass percentage (%)
1	1%PTCDA/Al_2O_3	1.4
2	5%PTCDA/Al_2O_3	5.0
3	10%PTCDA/Al_2O_3	7.2
4	15%PTCDA/Al_2O_3	13.2

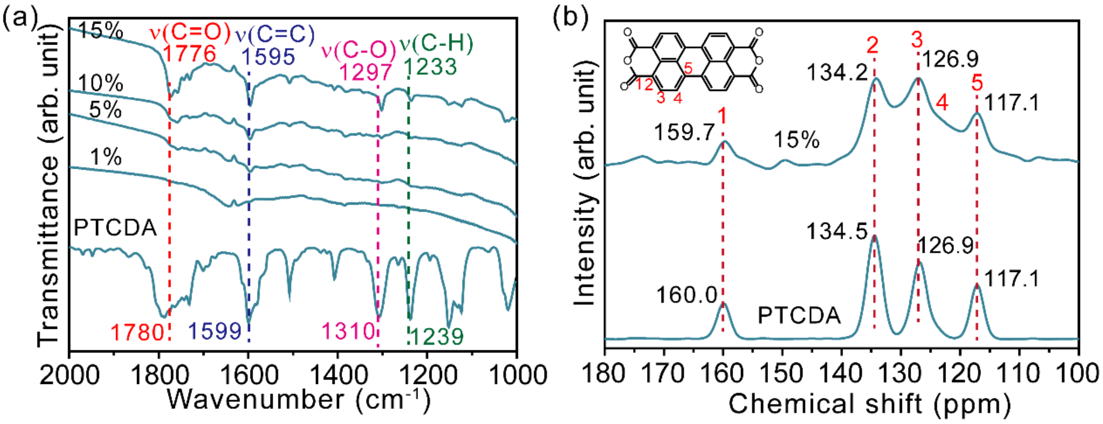

Supplementary Figure 3. Interaction between PTCDA and Al_2O_3. (a) FTIR spectra of PTCDA/Al_2O_3 with different PTCDA mass percentages. (b) Solid state ^{13}C NMR spectra of 15%PTCDA/Al_2O_3 and PTCDA.

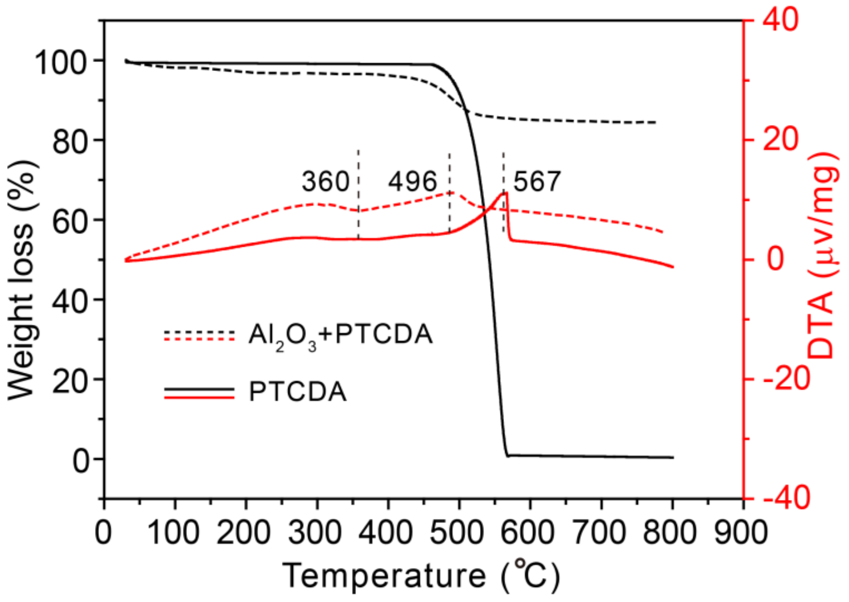

Supplementary Figure 4. Stability characterization of PTCDA. TG/DTA curves of PTCDA and the physical mixture of PTCDA and Al_2O_3.

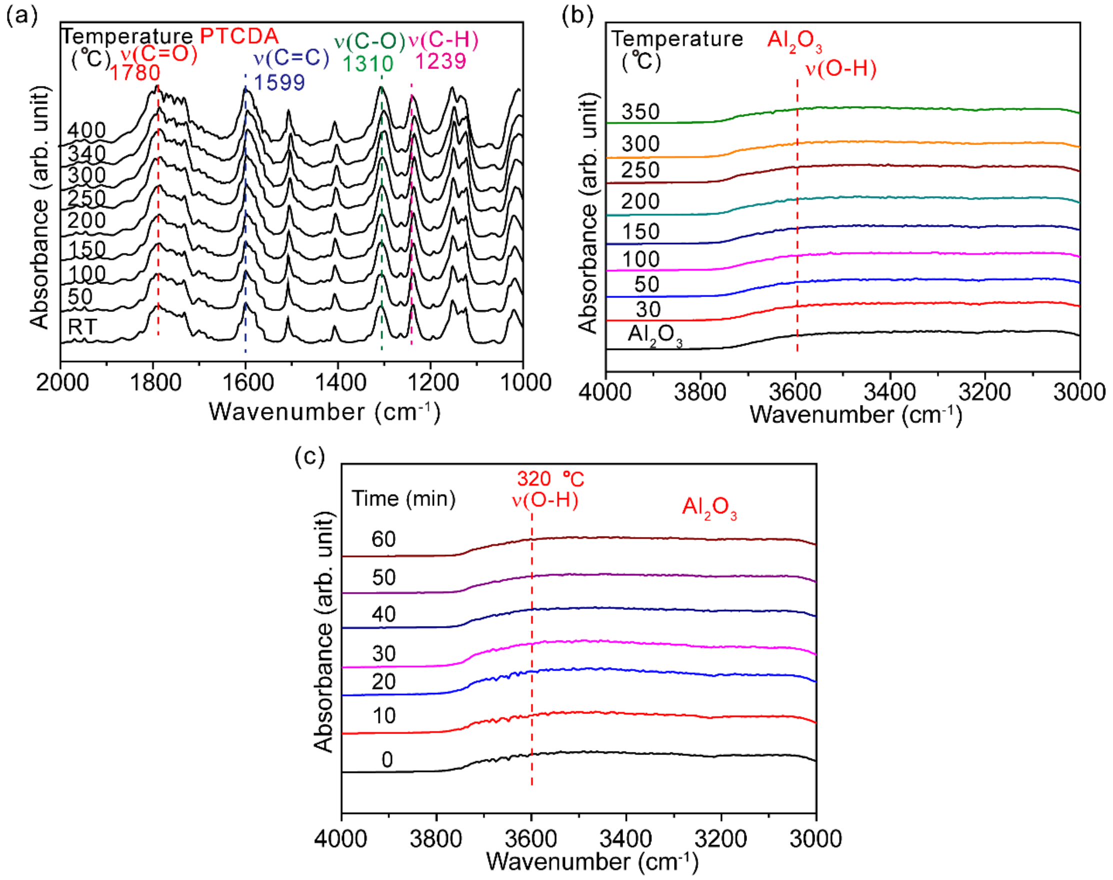

Supplementary Figure 5. *In-situ* DRIFTs. (a) *In-situ* DRIFTs of PTCDA calcined at different temperatures. (b-c) *In-situ* DRIFTs of Al_2O_3 calcined at (b) different temperatures and (c) at 320 °C for different time durations.

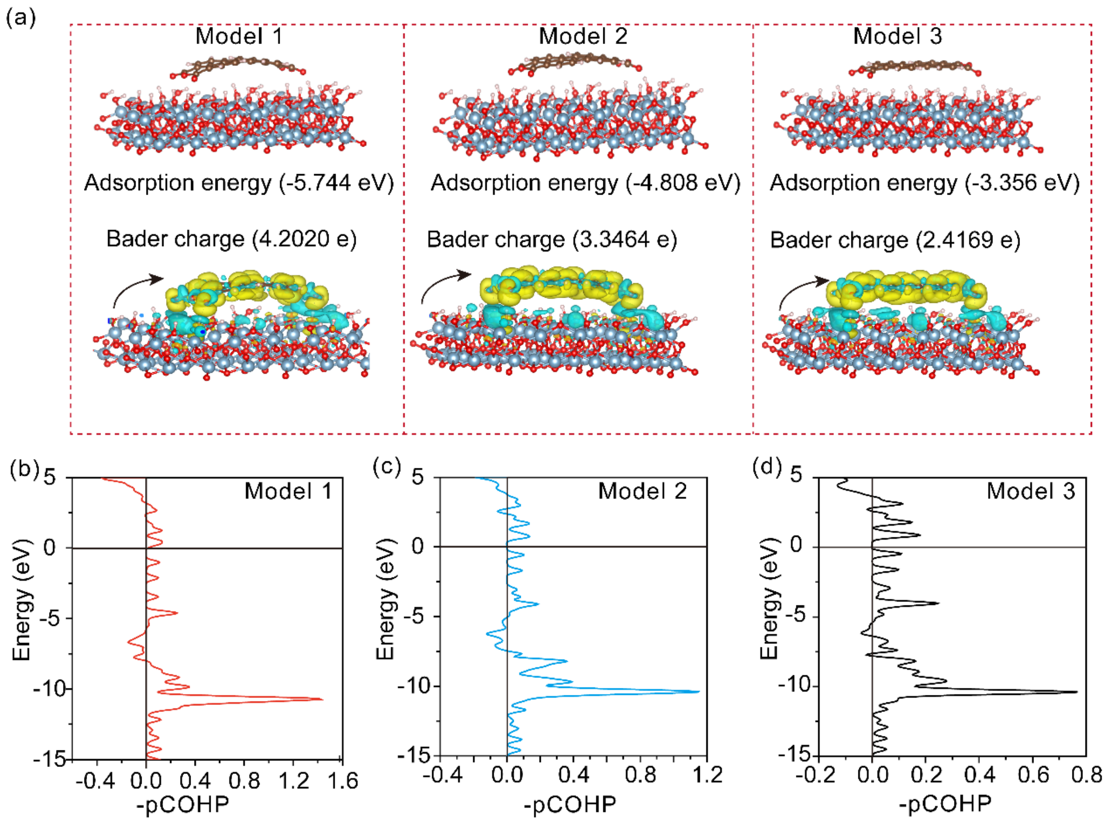

Supplementary Figure 6. Theoretical calculation. (a) Differential charge and Bader charge, and (b-d) negative-projected COHP (−pCOHP) for the O and H atoms with the shortest bond length in three different adsorption configurations for PTCDA/Al_2O_3(110).

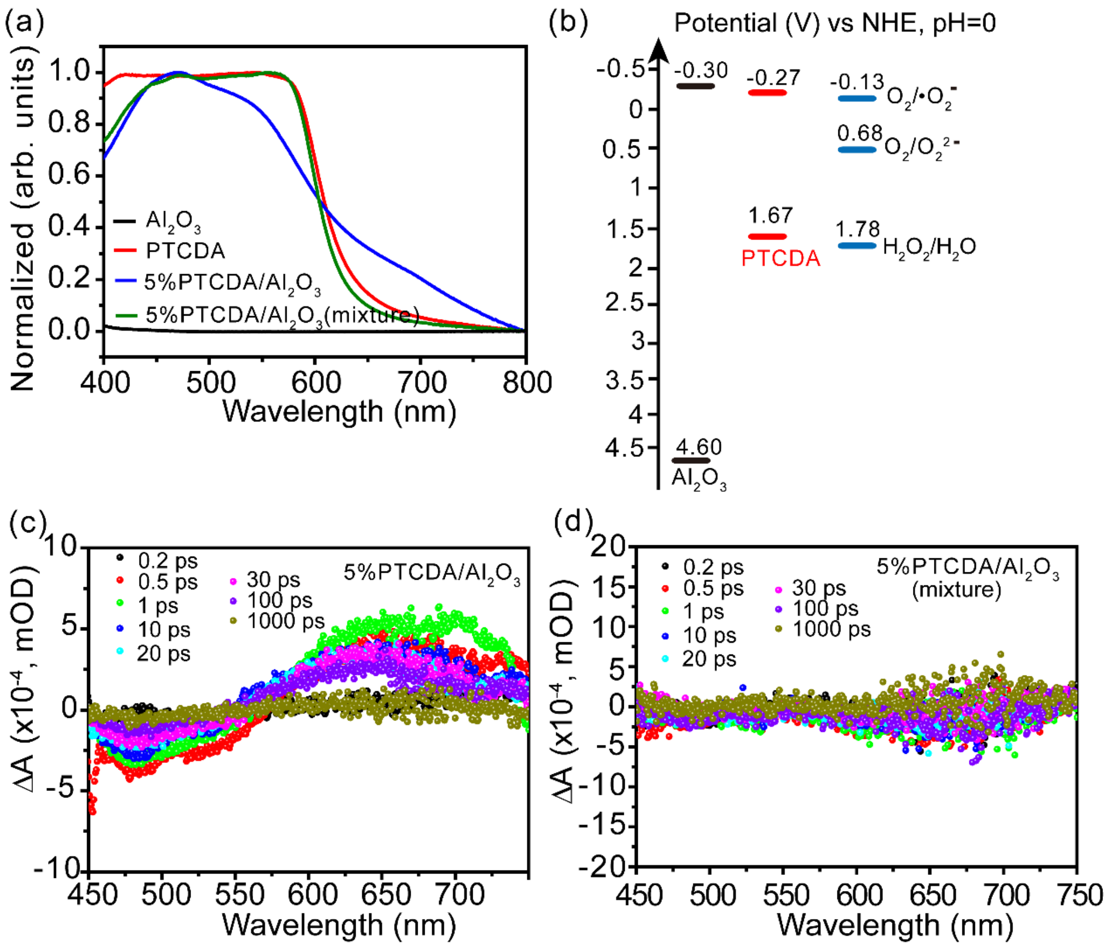

Supplementary Figure 7. Light absorption properties. (a) UV-vis absorption spectra of PTCDA, Al_2O_3, 5%PTCDA/Al_2O_3(physical mixture) and 5%PTCDA/Al_2O_3. (b) The typical redox potentials of PTCDA and Al_2O_3. (c-d) Time kinetics of femtosecond transient absorption spectra for (c) 5%PTCDA/Al_2O_3 and (d) 5%PTCDA/Al_2O_3(physical mixture) recorded in air.

Supplementary Table 2. Control experiments to study photocatalytic bromination reaction.

Entry	Photocatalysts	Time (h)	Atmosphere	Yield of *p*-bromoanisole (%)
1	5%PTCDA/Al_2O_3	5	Ar	0
2	5%PTCDA/Al_2O_3	5	O_2	95
3	5%PTCDA/Al_2O_3	5	Air	43
4	No	5	O_2	0
5[a]	H_2O_2	2	O_2	96
6[b]	HBrO	5	Air	95
7	No	5	Air	0

Reaction conditions: temperature: 30 °C; time: 5 h; acetonitrile: 1 mL; anisole: 0.2 mmol; HBr: 0.2 mL; blue LED: 100 mW cm^{-2}. [a] 0.25 mmol of H_2O_2 is added in the reaction system under O_2 atmosphere. [b] 0.25 mmol of HBrO is added in the reaction system under air atmosphere without HBr.

Supplementary Table 3. Evaluation of photocatalytic H_2O_2 production.

Entry	Photocatalysts	Amount of H_2O_2 (mmol)
1	5%PTCDA/Al_2O_3	0.009
2	PTCDA	0
3	5%PTCDA/Al_2O_3(physical mixture)	0
4[a]	5%PTCDA/Al_2O_3	0

Reaction conditions: photocatalyst: 10 mg; temperature: 30 °C; time: 5 h; acetonitrile: 1 mL; methanol: 0.5 mL; O_2: 1 atm; blue LED: 100 mW cm^{-2}. H_2O_2 is quantified by iodometry method.[2] [a] 0.2 mmol of H_2PtCl_6 is added in the reaction system under Ar atmosphere.

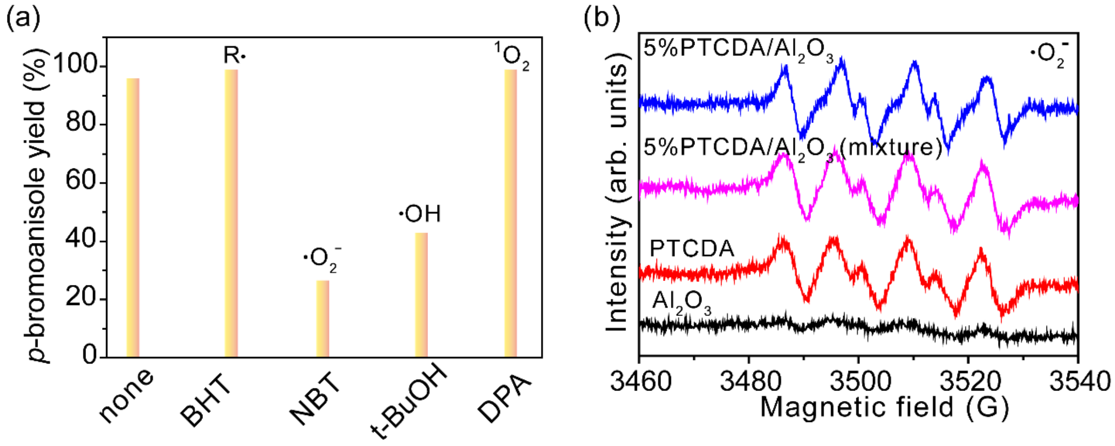

Supplementary Figure 8. Photocatalytic reaction mechanism. (a) The influence of different scavengers on the photocatalytic bromination reaction over 5%PTCDA/Al_2O_3. The amount of butylated hydroxytoluene (BHT), nitrotetrazolium blue chloride (NBT), *t*-butyl alcohol (*t*-BuOH) and 9,10-diphenylanthrene (DPA) used are 0.2 mmol. (b) EPR signals recorded over Al_2O_3, PTCDA, 5%PTCDA/Al_2O_3 and 5%PTCDA/Al_2O_3(physical mixture) with 5,5-dimethyl-L-pyrroline-N-oxide (DMPO) addition under visible light irradiation.

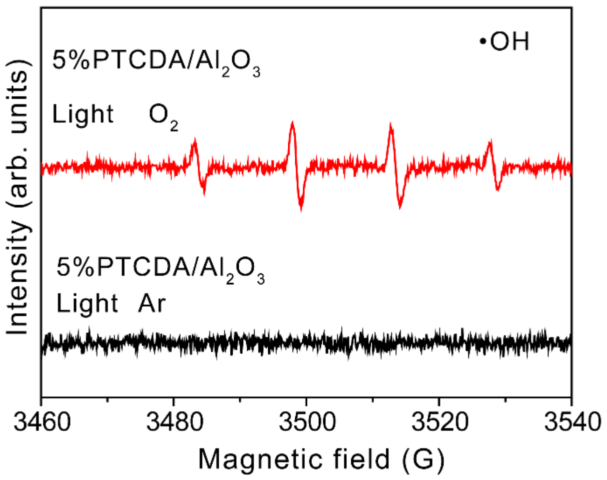

Supplementary Figure 9. Hydroxyl radical quantification. EPR signals recorded over 5%PTCDA/Al_2O_3 with 5,5-dimethyl-L-pyrroline-N-oxide (DMPO) addition in H_2O solution with Ar or O_2 bubbling under visible light irradiation.

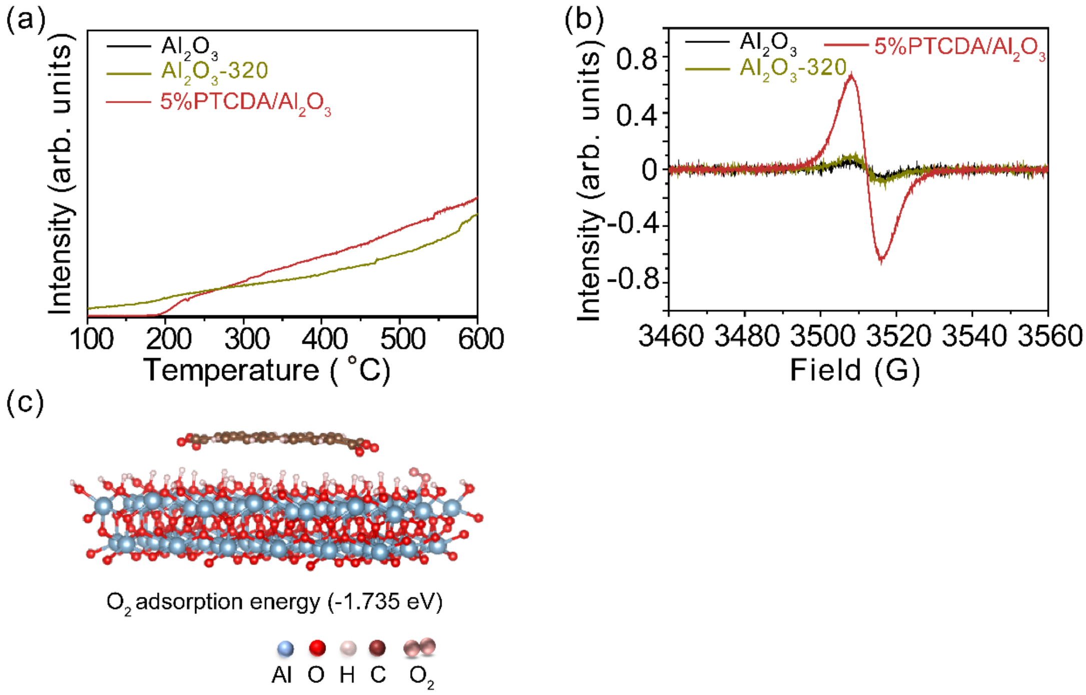

Supplementary Figure 10. Oxygen vacancy quantification and adsorption mode of O_2 on PTCDA/Al_2O_3(physical mixture). (a) O_2-TPD and (b) EPR spectra of Al_2O_3, Al_2O_3-320 (Al_2O_3 calcined at 320 °C for 1 h) and 5%PTCDA/Al_2O_3. (c) The adsorption and activation mode of O_2 on the surface of Model 3.

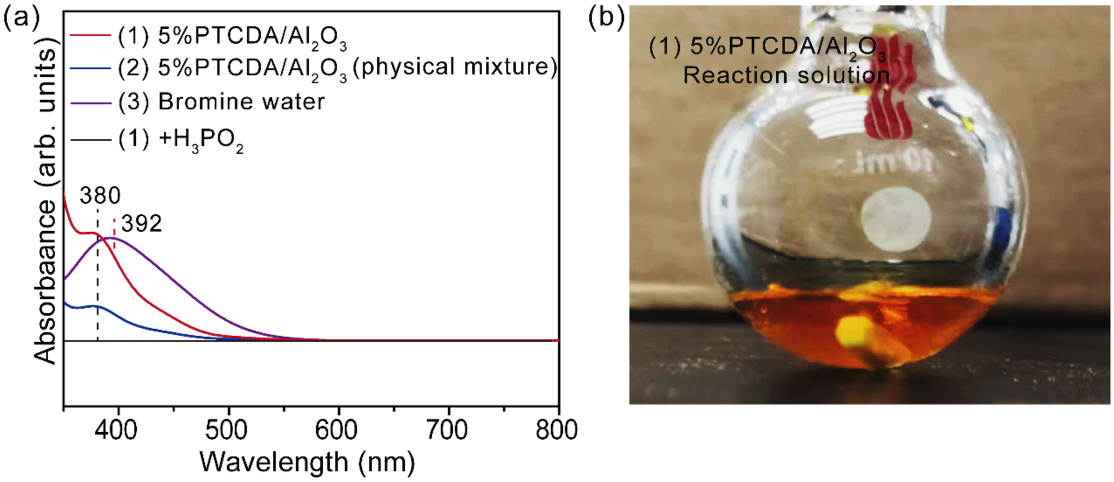

Supplementary Figure 11. Br_2 detection. (a) UV-vis spectra of the solutions for photocatalytic reaction (reaction solution under light with acetonitrile as solvent, 0.00843 mol/L of bromine water and reaction solution under light with acetonitrile as solvent and 10 µL of H_3PO_2 as reducing agent of Br_2 to Br^-. (b) The reaction solution under light with 5%PTCDA/Al_2O_3. Reaction conditions: photocatalyst: 10 mg; temperature: 30 °C; time: 5 h; acetonitrile: 1 mL; HBr: 0.2 mL; Ar: 1 atm; H_2PtCl_6: 0.2 mmol; blue LED: 100 mW cm^{-2}.

2. NMR Spectra

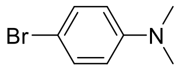

4-Bromo-N,N-dimethylaniline, 89%.[3] ^{1}H NMR (400 MHz, $CDCl_3$) δ 7.29 (d, *J* = 9.1 Hz, 2H), 6.60 (d, *J* = 9.1 Hz, 2H), 2.92 (s, 6H).

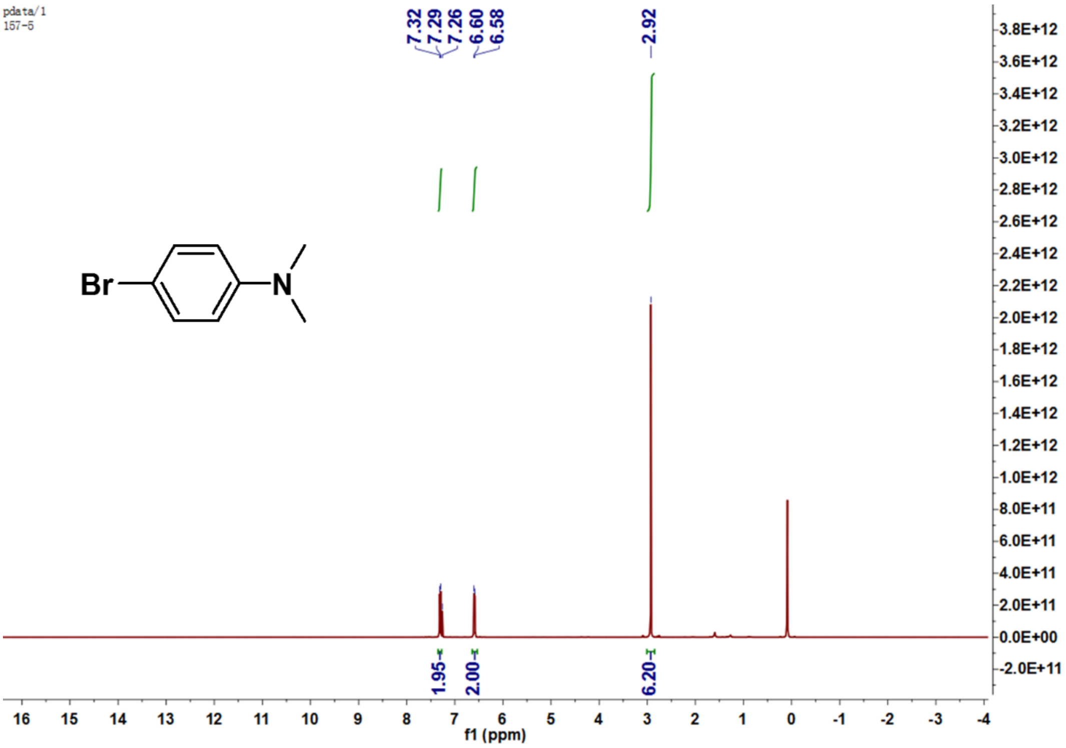

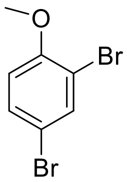

1,4-Dibromo-2-methoxybenzene, 72%.[4] ^{1}H NMR (600 MHz, $CDCl_3$) δ 7.66 (d, *J* = 2.4 Hz, 1H), 7.38 (dd, *J* = 8.8, 2.4 Hz, 1H), 6.77 (d, *J* = 8.8 Hz, 1H), 3.87 (s, 3H). ^{13}C NMR (150 MHz, $CDCl_3$) δ 155.3, 135.5, 131.3, 113.2, 112.9, 112.6, 56.5.

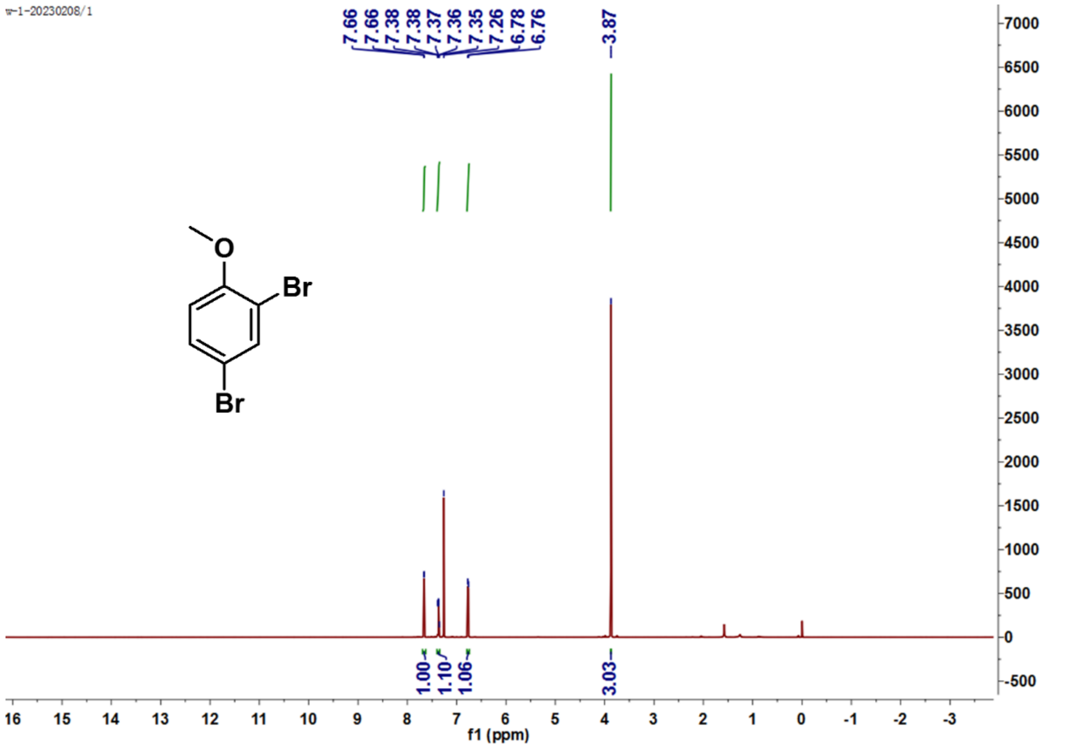

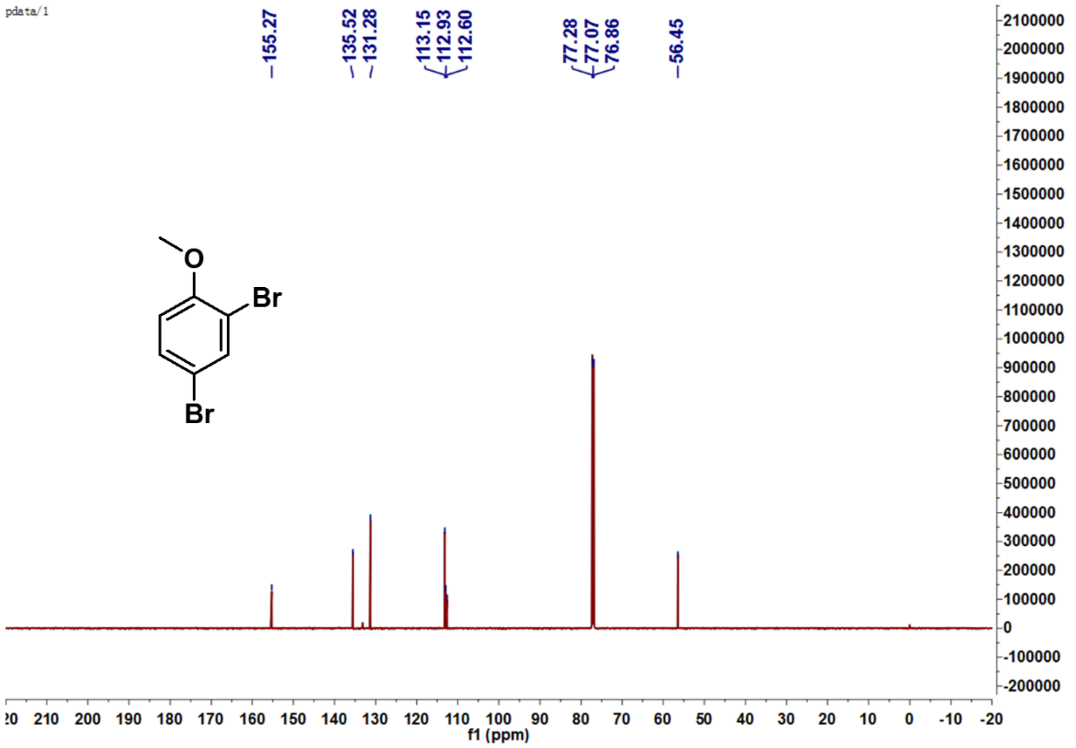

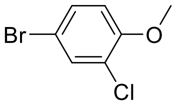

4-Bromo-2-chloro-1-methoxybenzene, 88%.[5] ^{1}H NMR (400 MHz, $CDCl_3$) δ 7.49 (s, 1H), 7.32 (d, *J* = 8.7 Hz, 1H), 6.79 (d, *J* = 8.8 Hz, 1H), 3.88 (s, 3H).

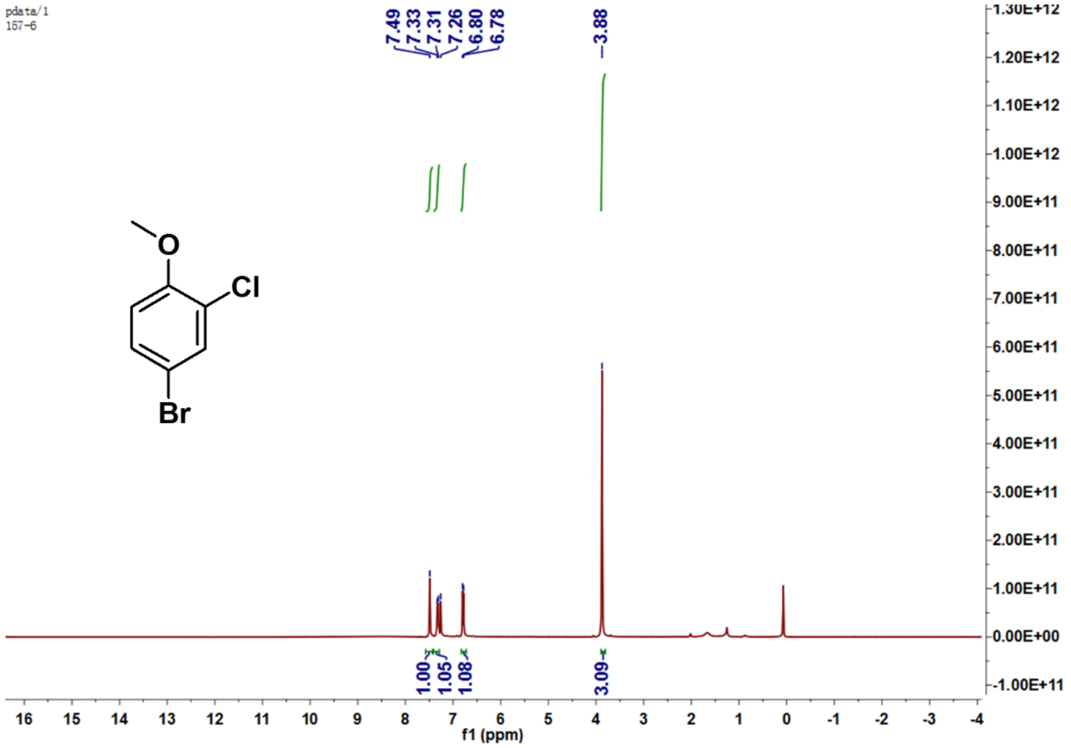

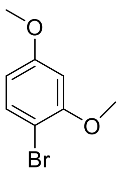

1-Bromo-2,4-dimethoxybenzene, 87%.[6] ^{1}H NMR (500 MHz, $CDCl_3$) δ 7.40 (d, *J* = 8.7 Hz, 1H), 6.49 (d, *J* = 2.7 Hz, 1H), 6.39 (dd, *J* = 8.7, 2.7 Hz, 1H), 3.86 (s, 3H), 3.79 (s, 3H).

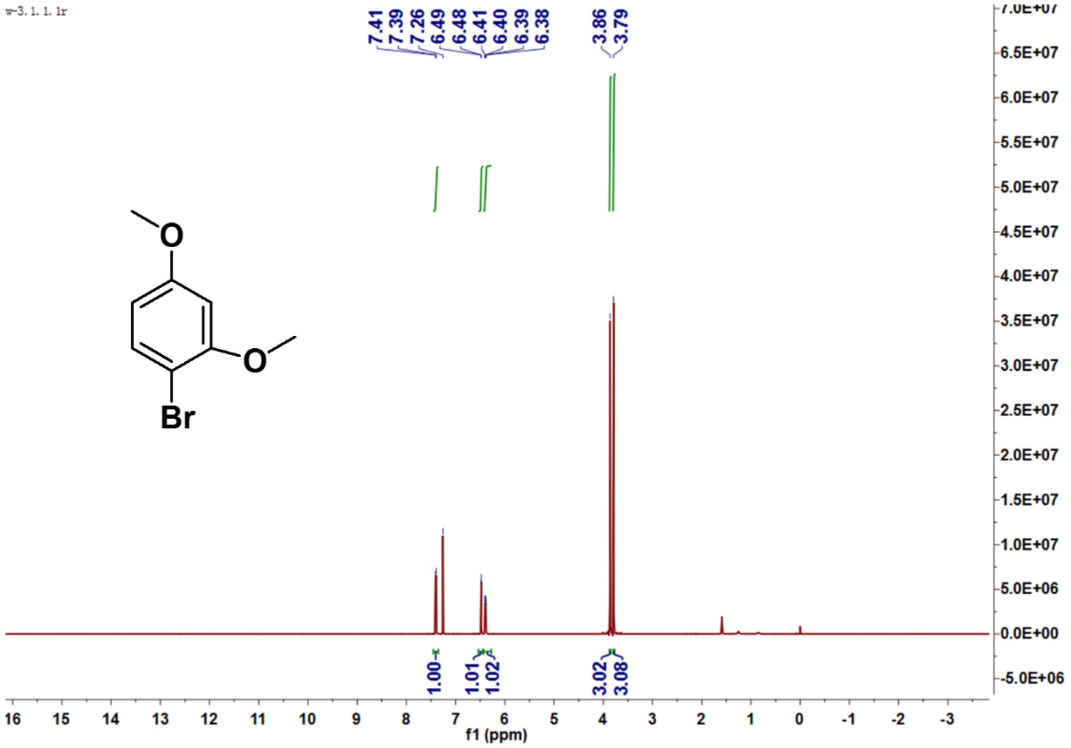

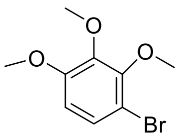

1,2,3-Trimethoxybromobenzene, 92%.[6] ^{1}H NMR (500 MHz, $CDCl_3$) δ 7.20 (d, *J* = 8.9 Hz, 1H), 6.58 (d, *J* = 9.0 Hz, 1H), 3.90 (s, 3H), 3.88 (s, 3H), 3.84 (s, 3H).

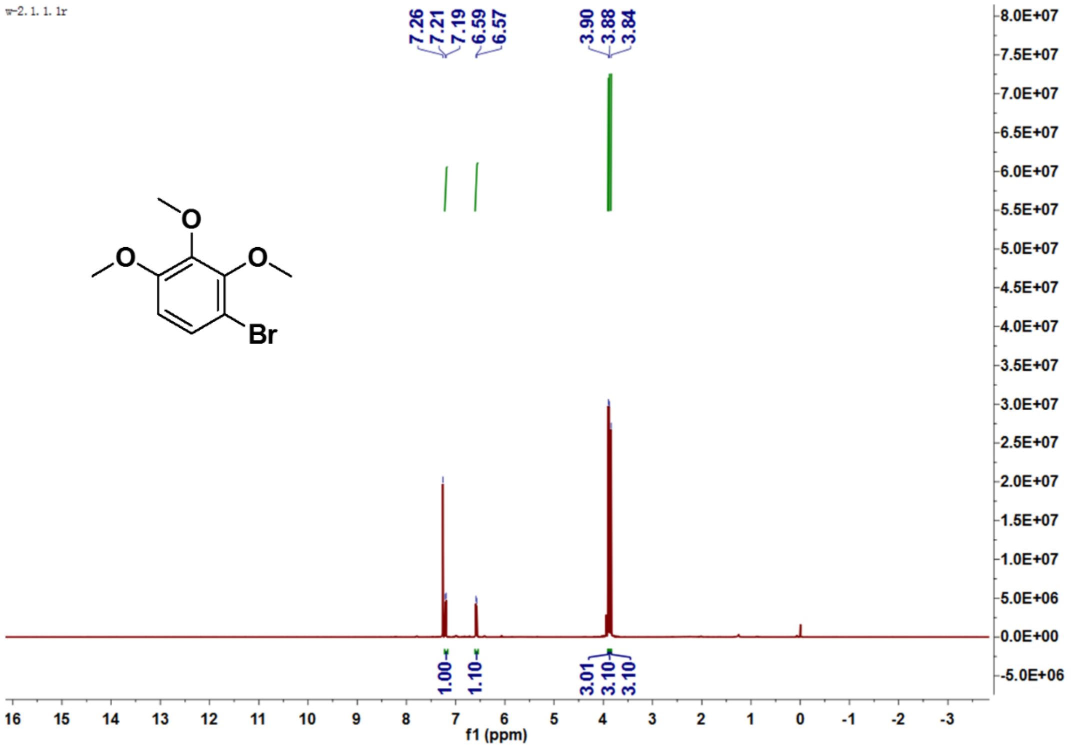

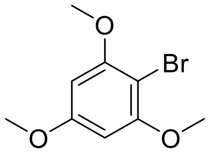

2,4,6-Trimethoxybromobenzene, 80%.[7] ^{1}H NMR (600 MHz, $CDCl_3$) δ 6.14 (s, 2H), 3.84 (s, 6H), 3.79 (s, 3H).

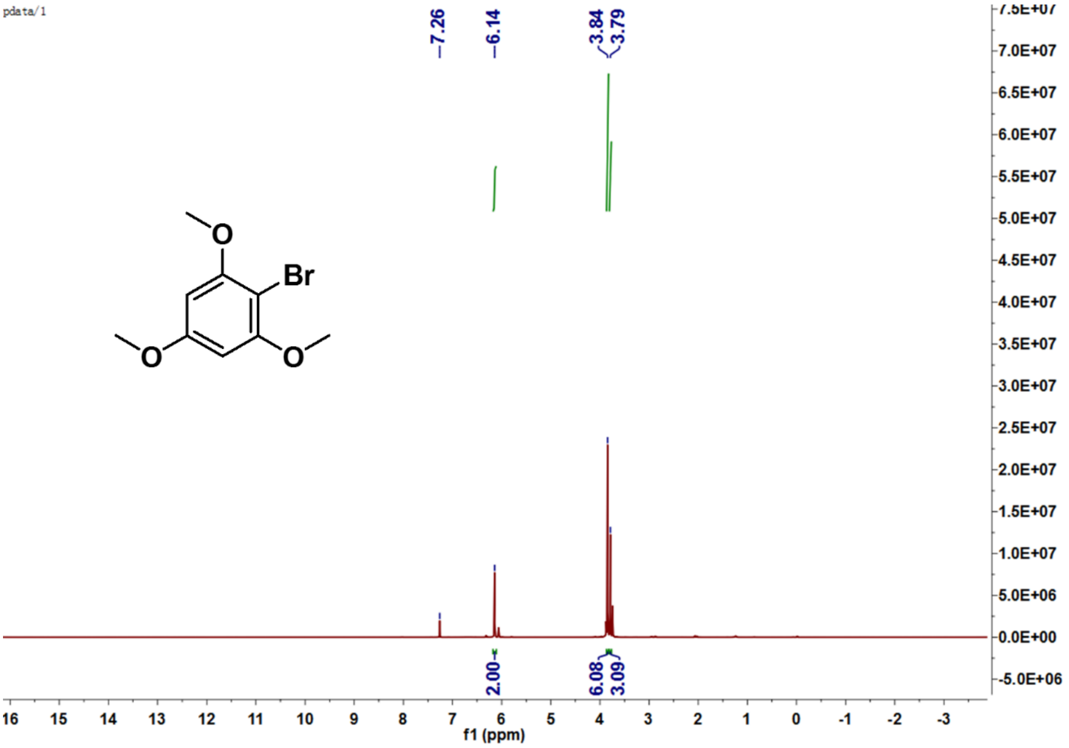

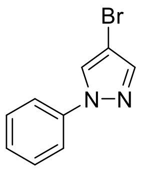

4-Bromo-1-phenyl-pyrazole, 71%.[8] ^{1}H NMR (500 MHz, $CDCl_3$) δ 7.93 (s, 1H), 7.67 (s, 1H), 7.64 (dd, *J* = 8.5, 0.9 Hz, 2H), 7.45 (t, *J* = 8.0 Hz, 2H), 7.31 (t, *J* = 7.4 Hz, 1H). ^{13}C NMR (125 MHz, $CDCl_3$) δ 141.5, 139.6, 129.6, 127.1, 127.03 (s), 119.0, 95.6.

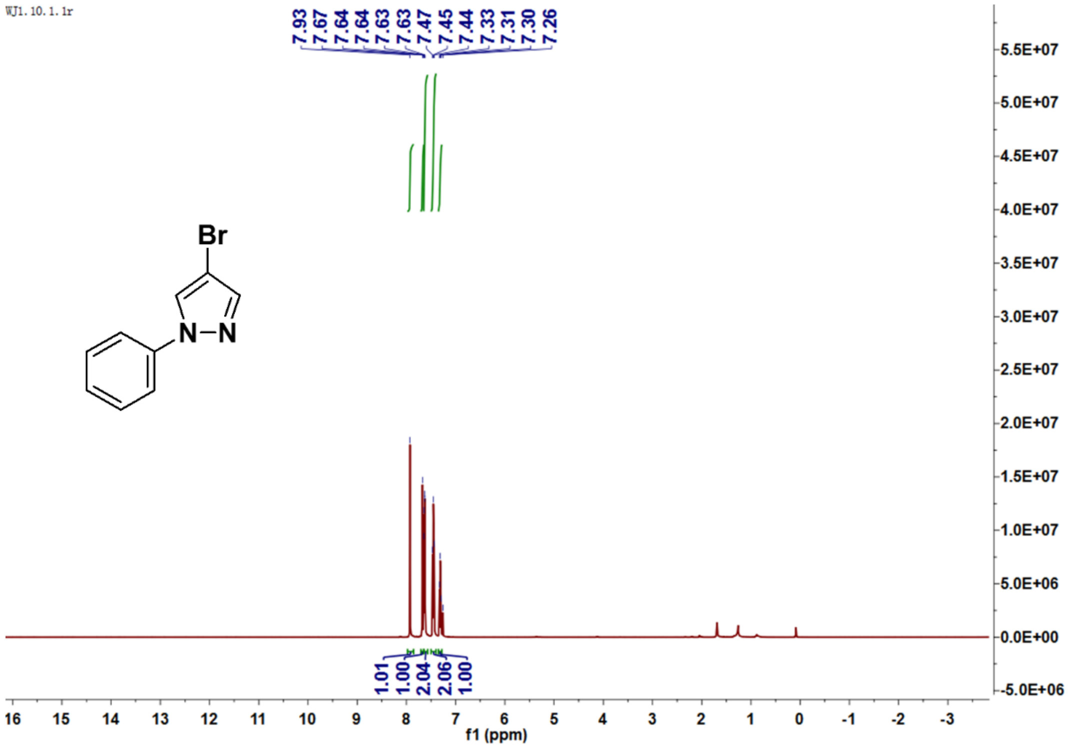

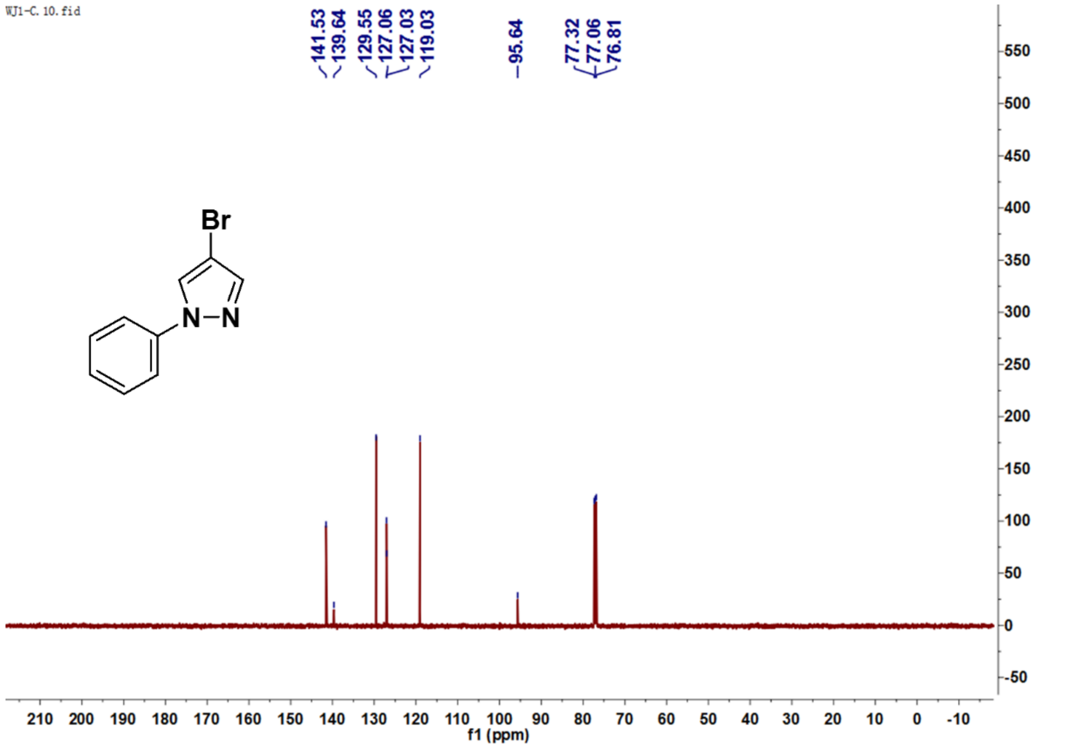

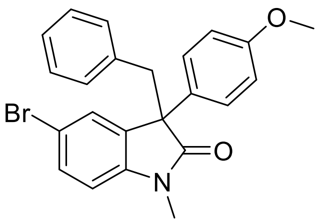

3-Benzyl-5-bromo-3-(4-methoxyphenyl)-1-methylindolin-2-one, 92%. ^{1}H NMR (600 MHz, $CDCl_3$) δ 7.38 (d, *J* = 8.9 Hz, 2H), 7.33 (dd, *J* = 8.2, 1.9 Hz, 1H), 7.30 (d, *J* = 1.9 Hz, 1H), 7.09 – 7.03 (m, 3H), 6.9 (d, *J* = 8.9 Hz, 2H), 6.84 (d, *J* = 6.6 Hz, 2H), 6.48 (d, *J* = 8.2 Hz, 1H), 3.80 (s, 3H), 3.66 (d, *J* = 12.9 Hz, 1H), 3.40 (d, *J* = 12.8 Hz, 1H), 2.92 (s, 3H). ^{13}C NMR (150 MHz, $CDCl_3$) δ 177.6, 159.1, 142.8, 135.3, 133.8, 131.0, 129.9, 128.5, 128.3, 127.6, 126.8, 114.9, 114.2, 109.5, 57.9, 55.4, 44.1, 26.2. HRMS (ESI) Calcd for $C_{23}H_{20}BrNO_2$ $[M+Na]^+$: 444.0570; Found: 444.0581.

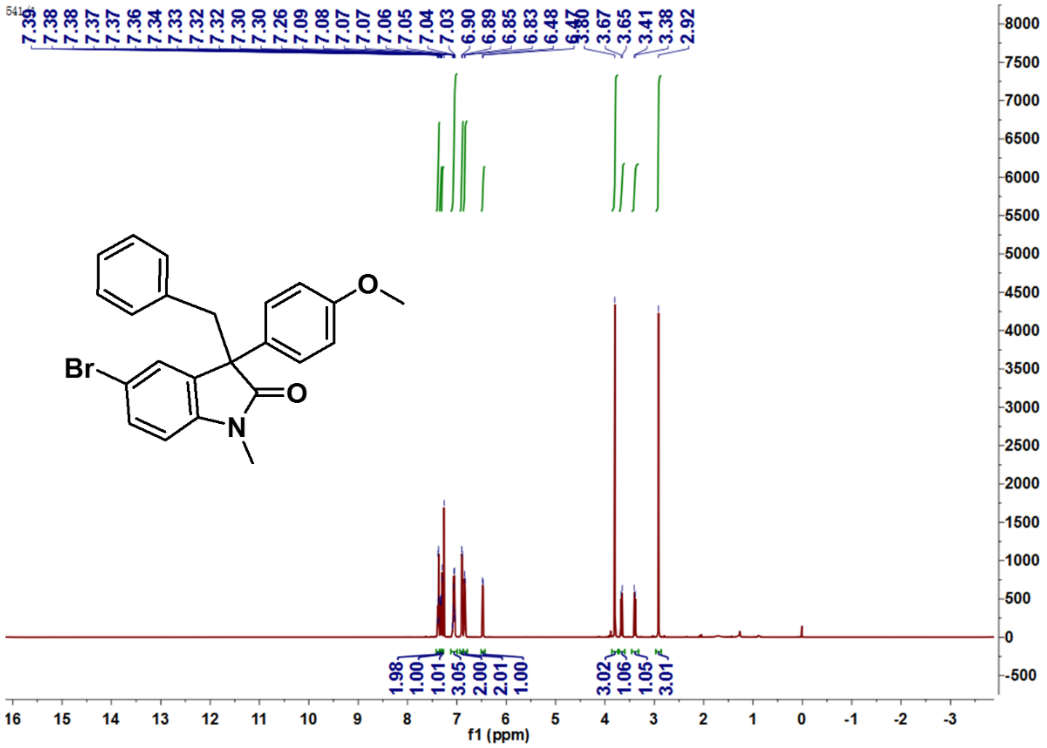

142 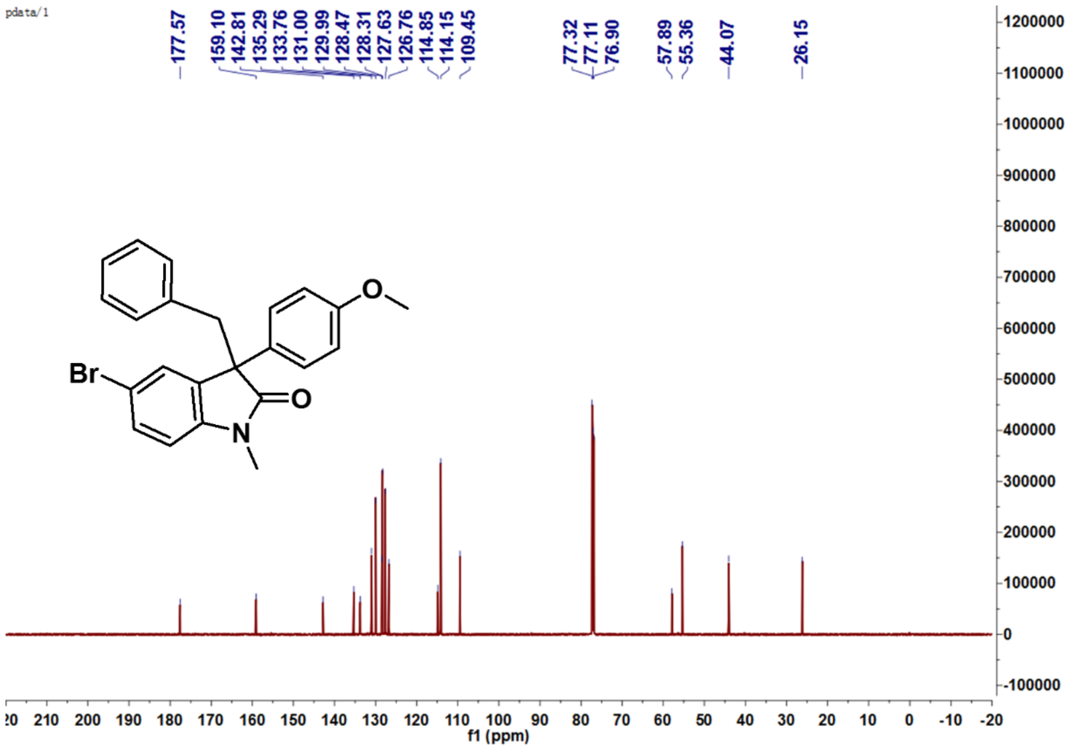

pdata/1
177.57
159.10
142.81
135.29
133.76
131.00
129.99
128.47
128.31
127.63
126.76
114.85
114.15
109.45
77.32
77.11
76.90
57.89
55.36
44.07
26.15
Br
O
N
f1 (ppm)

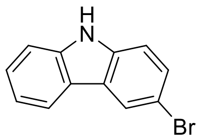

3-Bromo-9H-carbazole, 92%.[3] ^{1}H NMR (400 MHz, DMSO) δ 11.44 (s, 1H), 8.36 (d, *J* = 1.7 Hz, 1H), 8.16 (d, *J* = 7.8 Hz, 1H), 7.56 – 7.46 (m, 3H), 7.43 (t, *J* = 8.1 Hz, 1H), 7.18 (t, *J* = 7.8 Hz, 1H). ^{13}C NMR (100 MHz, DMSO) δ 140.6, 138.9, 128.3, 126.8, 124.9, 123.2, 121.9, 121.2, 119.4, 113.4, 111.7, 111.0.

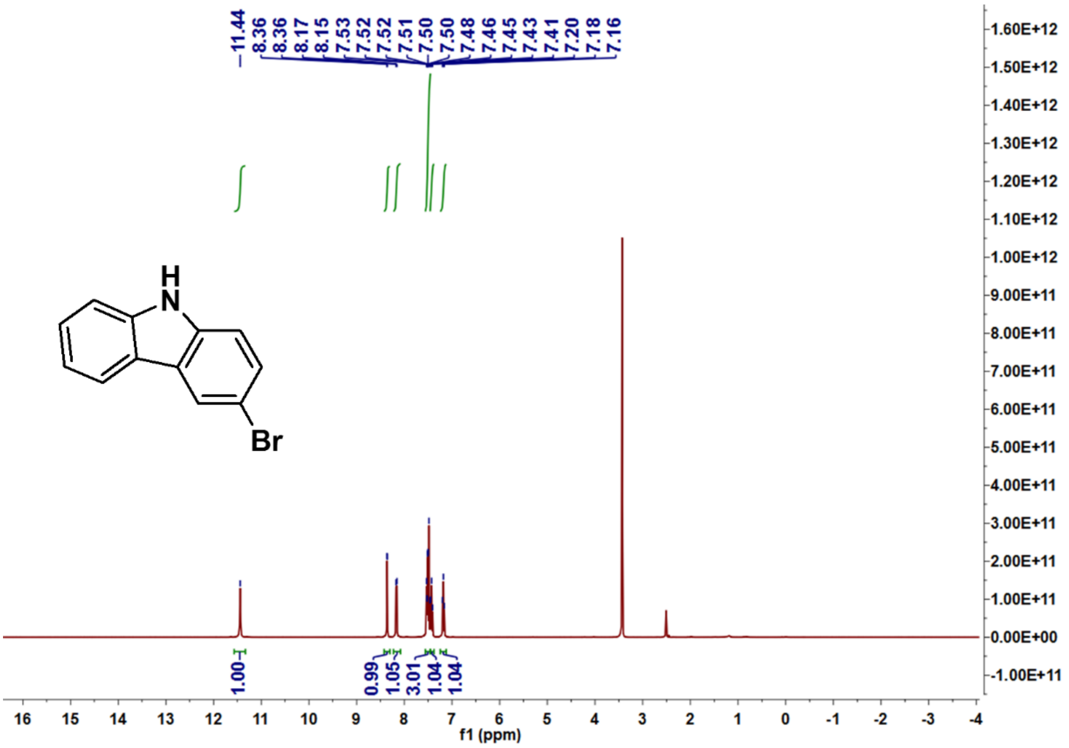

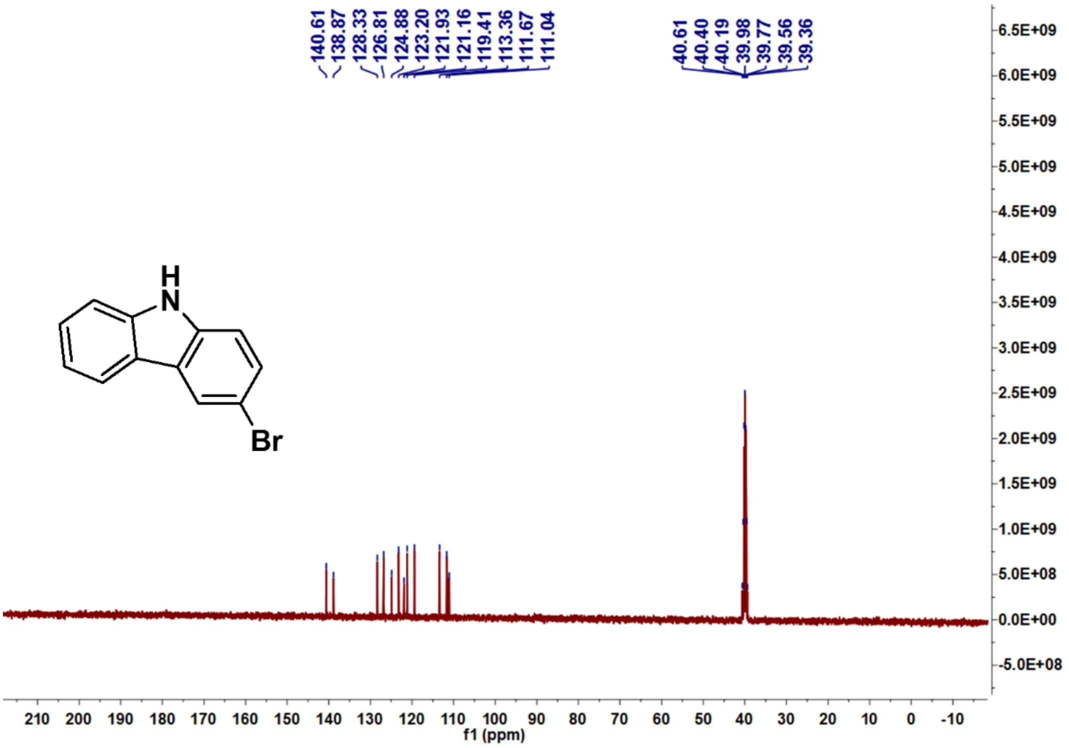

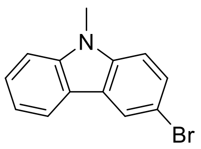

3-Bromo-9-methylcarbazole, 83%.[3] ^{1}H NMR (600 MHz, $CDCl_3$) δ 8.23 (s, 1H), 8.08 (d, *J* = 7.8 Hz, 1H), 7.59 (d, *J* = 8.6 Hz, 1H), 7.55 (t, *J* = 7.7 Hz, 1H), 7.43 (d, *J* = 8.2 Hz, 1H), 7.31 (d, *J* = 7.0 Hz, 1H), 7.29 (d, *J* = 8.4 Hz, 1H), 3.84 (s, 3H). ^{13}C NMR (150 MHz, $CDCl_3$) δ 141.3, 139.6, 128.3, 126.4, 124.5, 123.0, 121.7, 120.5, 119.3, 111.7, 109.9, 108.7, 29.2.

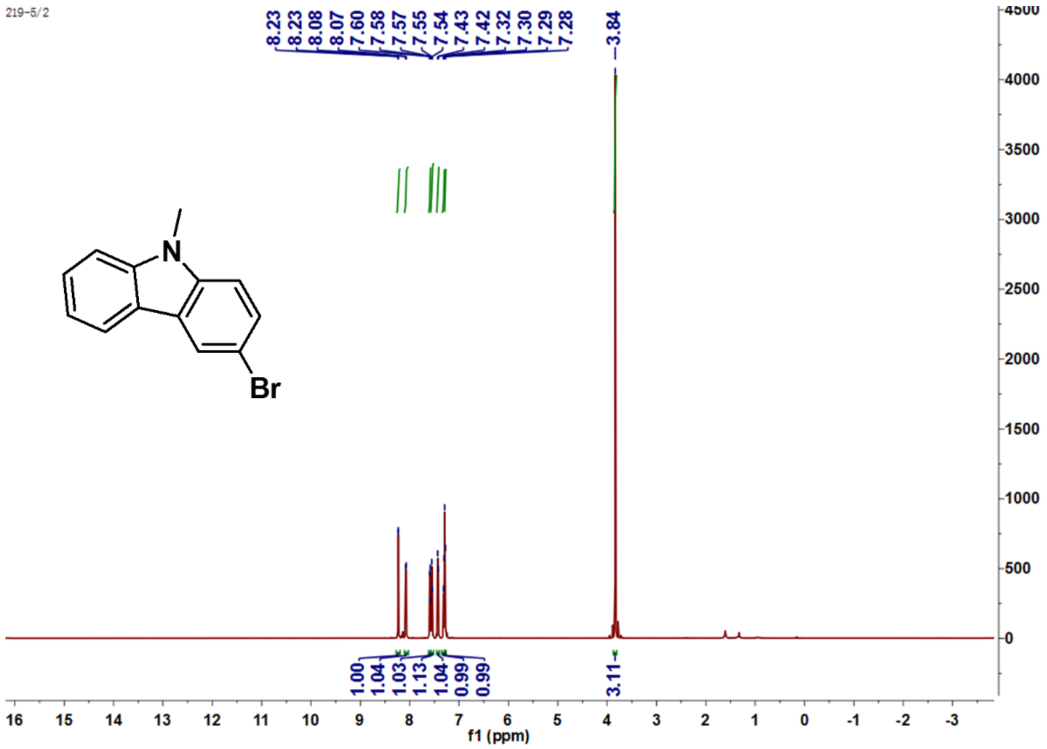

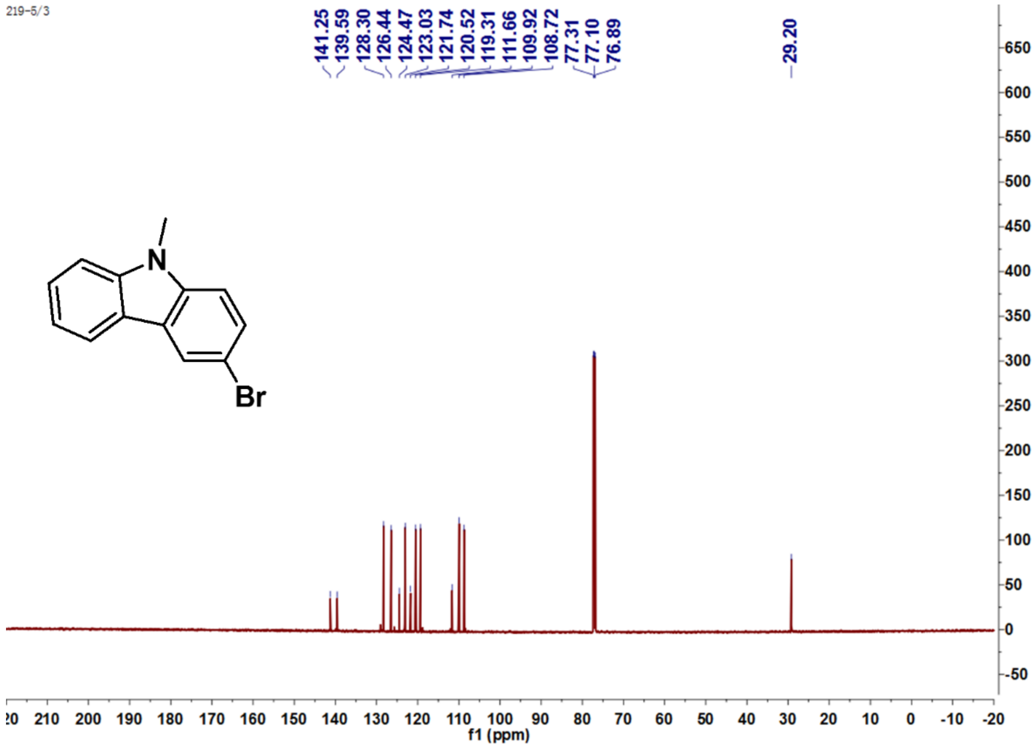

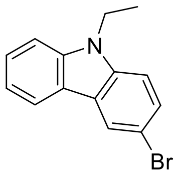

3-Bromo-9-ethylcarbazole, 83%.[9] ^{1}H NMR (600 MHz, $CDCl_3$) δ 8.23 (d, *J* = 1.8 Hz, 1H), 8.07 (d, *J* = 7.8 Hz, 1H), 7.57 (dd, *J* = 8.6, 1.9 Hz, 1H), 7.53 – 7.51 (m, 1H), 7.43 (d, *J* = 8.2 Hz, 1H), 7.30 (d, *J* = 8.6 Hz, 1H), 7.28 (t, *J* = 7.5 Hz, 1H), 4.35 (d, *J* = 7.3 Hz, 2H), 1.44 (t, *J* = 7.3 Hz, 3H). ^{13}C NMR (150 MHz, $CDCl_3$) δ 140.2, 138.6, 128.3, 126.4, 124.7, 123.2, 121.9, 120.7, 119.3, 111.6, 109.9, 108.7, 37.7, 13.8.

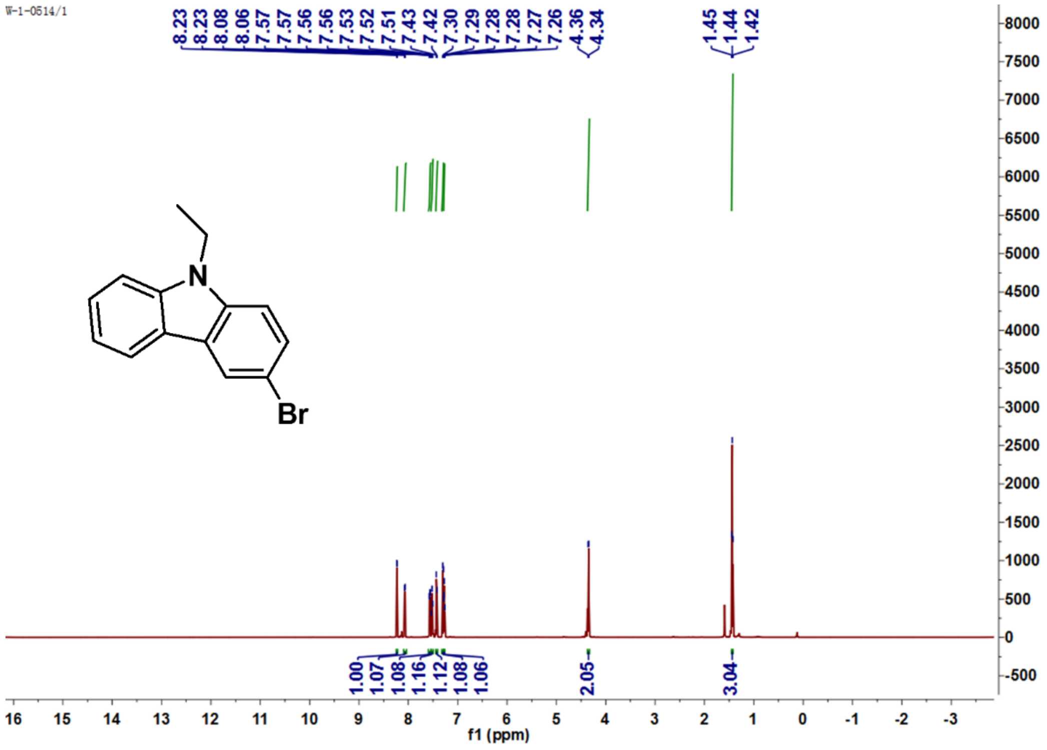

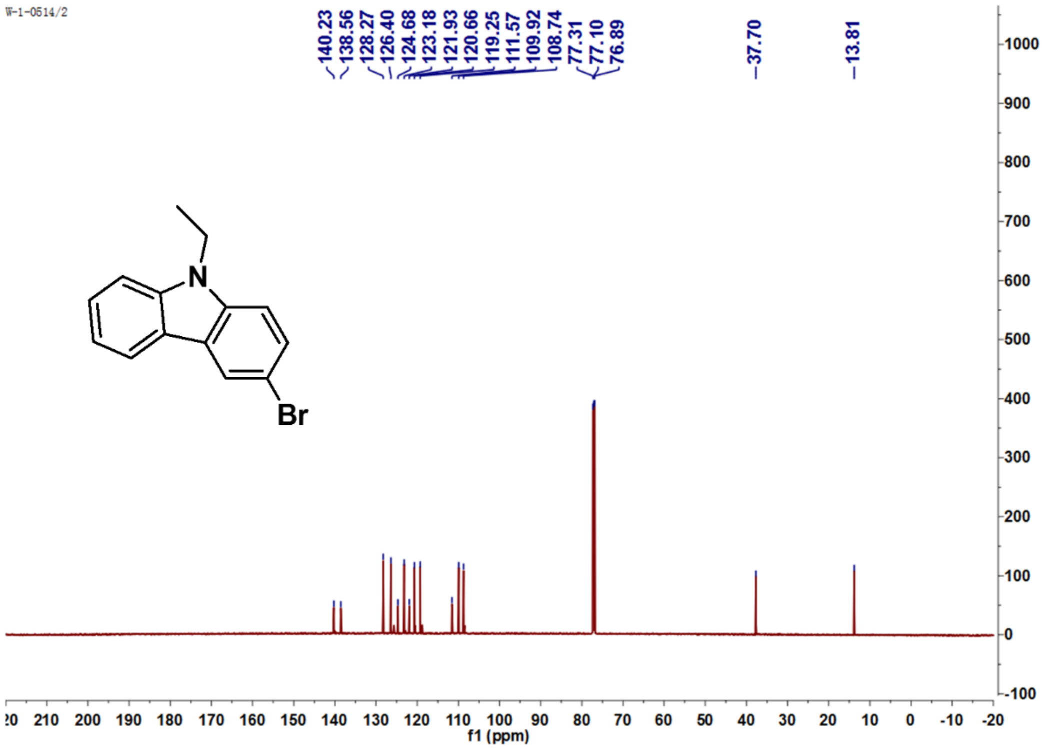
W-1-0514/2
140.23
138.56
128.27
126.40
124.68
123.18
121.93
120.66
119.25
111.57
109.92
108.74
77.31
77.10
76.89
37.70
13.81
N
Br
f1 (ppm)

3. Supplementary References

1 Guo, Y. *et al.* Perylenetetracarboxylic acid nanosheets with internal electric fields and anisotropic charge migration for photocatalytic hydrogen evolution. *Nat. Commun.* **13**, 2067, (2022).

2 Zhang, Y.-Z., Liang, C., Feng, H.-P. & Liu, W. Nickel single atoms anchored on ultrathin carbon nitride for selective hydrogen peroxide generation with enhanced photocatalytic activity. *Chem. Engin. J.* **446**, 137379, (2022).

3 Dalai, P. G., Palit, K. & Panda, N. Generation of dimethyl dulfoxide coordinated thermally stable halogen cation pools for C−H halogenation. *Adv. Synth. Catal.* **364**, 1031-1038, (2022).

4 Quibell, Jacob M., Perry, G. J. P., Cannas, D. M. & Larrosa, I. Transition-metal-free decarboxylative bromination of aromatic carboxylic acids. *Chem. Sci.* **9**, 3860-3865, (2018).

5 Mostafa, M. A. B., Bowley, R. M., Racys, D. T., Henry, M. C. & Sutherland, A. Iron(III)-catalyzed chlorination of activated arenes. *J. Org. Chem.* **82**, 7529-7537, (2017).

6 Ren, Y. L., Wang, B., Tian, X. Z., Zhao, S. & Wang, J. Aerobic oxidative bromination of arenes using an ionic liquid as both the catalyst and the solvent. *Tetrahedron Lett.* **56**, 6452-6455, (2015).

7 Li, R. *et al.* Photocatalytic selective bromination of electron-rich aromatic compounds using microporous organic polymers with visible light. *ACS Catal.* **6**, 1113-1121, (2016).

8 Samanta, R. C. & Yamamoto, H. Selective halogenation using an aniline catalyst. *Chem. Eur. J.* **21**, 11976-11979, (2015).

9 Przypis, L. & Walczak, K. Z. Copper(II)-catalyzed iodinations of carbazoles: access to functionalized carbazoles. *J. Org. Chem.* **84**, 2287-2296, (2019).
